# Multidelay ASL of the pediatric brain

**DOI:** 10.1259/bjr.20220034

**Published:** 2022-05-12

**Authors:** Xavier Golay, Mai-Lan Ho

**Affiliations:** MR Neurophysics and Translational Neuroscience, UCL Queen Square Institute of Neurology London, London, England, UK; Radiology, Nationwide Children’s Hospital and The Ohio State University, Columbus, OH, USA

## Abstract

Arterial spin labeling (ASL) is a powerful noncontrast MRI technique for evaluation of cerebral blood flow (CBF). A key parameter in single-delay ASL is the choice of postlabel delay (PLD), which refers to the timing between the labeling of arterial free water and measurement of flow into the brain. Multidelay ASL (MDASL) utilizes several PLDs to improve the accuracy of CBF calculations using arterial transit time (ATT) correction. This approach is particularly helpful in situations where ATT is unknown, including young subjects and slow-flow conditions. In this article, we discuss the technical considerations for MDASL, including labeling techniques, quantitative metrics, and technical artefacts. We then provide a practical summary of key clinical applications with real-life imaging examples in the pediatric brain, including stroke, vasculopathy, hypoxic-ischemic injury, epilepsy, migraine, tumor, infection, and metabolic disease.

## Introduction

Arterial spin labeling (ASL) is a powerful noncontrast MRI technique for evaluation of cerebral blood flow (CBF). A key parameter in single-delay ASL is the choice of postlabel delay (PLD), which refers to the timing between the labeling of arterial free water and measurement of flow into the brain. Multidelay ASL (MDASL) utilizes several PLDs to improve the accuracy of CBF calculations using arterial transit time (ATT) correction. This approach is particularly helpful in situations where ATT is unknown, including young subjects and slow-flow conditions. In this article, we discuss the technical considerations for MDASL, including labeling techniques, quantitative metrics, and technical artefacts. We then provide a practical summary of key clinical applications with real-life imaging examples in the pediatric brain, including stroke, vasculopathy, hypoxic-ischemic injury, epilepsy, migraine, tumor, infection, and metabolic disease.

## Technical considerations

ASL is a non-contrast MRI technique used for the measurement of perfusion-related parameters.^1,2^ In its most simple incarnation, it uses a preparation sequence upstream from the tissue of interest to invert the inflowing arterial water spins, and measures its arrival downstream. The rate at which the labeled blood is delivered to the tissue is called cerebral blood flow (CBF), measured in standard units of mL/min/100g. A typical ASL measurement consists of two consecutive acquisitions, the first one performed following labeling of the water spins, and the second without such labeling.^3^ The subtraction of both acquisitions leads to a perfusion-weighted image, which needs further processing to be transformed into a quantitative CBF map. Importantly, the small inherent signal-to-noise ratio (SNR) of the method—a direct result of a subtraction between two acquisitions—can typically be compensated by using multiple repetitions or dedicated 3D-based acquisition methods, based on a combination of a rapid acquisition scheme (typically EPI or spiral imaging) with a fast-spin echo to rapidly cover large volumes.^3^

**Figure 1.**
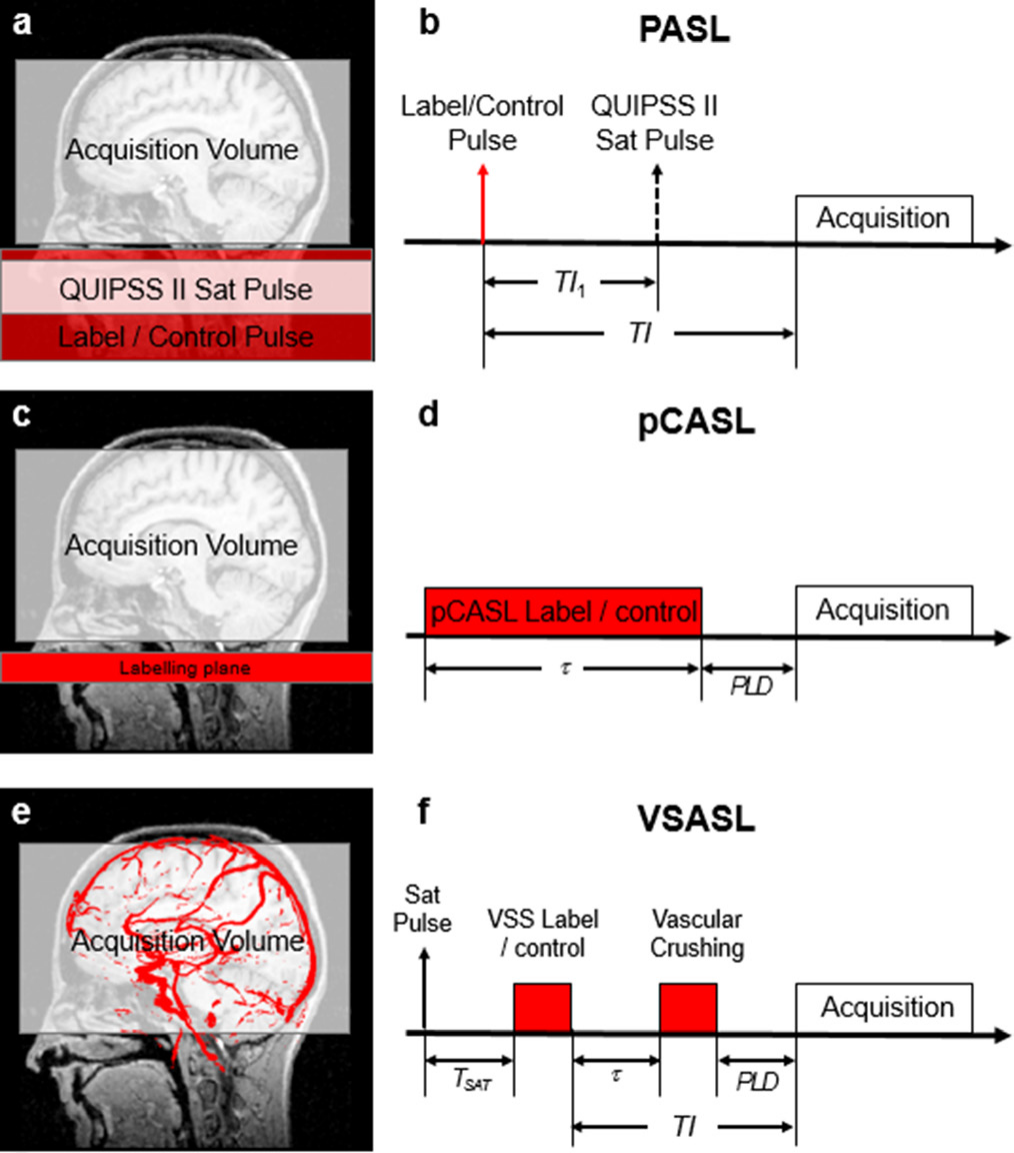
Geometrical representation (**a,c,e**) and pulse sequence diagrams (**b,d,f**) for various ASL labeling schemes. Labeling components of pulse sequences are shown in red. For PASL (**a**), labeling is volume selective and occurs in a single instant (**b**). The labeling duration is set by the additional saturation pulse applied so as to cut off the tail of the labeled blood bolus. The time *TI* is selected to allow the labeled spins to enter into the acquisition volume. For pCASL, the labeling volume is restricted to a thick plane (**c**), applied over a long labeling duration *t* (**d**). A corresponding postlabel delay (*PLD*) is included to allow the labeled spins to clear the intravascular space. For velocity-selective ASL (VSASL), the pulse sequence includes a saturation pulse, and two labeling modules (also applied over a very short amount of time), which are non-spatially selective, but will select all the flowing spins (red in (**e**)). These can then be detected after a post-labelling delay (PLD) (**f**), in a way similar to pCASL (**d**).

There are several ways to perform labeling of the arterial spins, and we will discuss the three most promising techniques in the brain. The first and most intuitive approach is called pulsed arterial spin labeling or PASL, and consists of positioning a large inversion volume upstream from the tissue of the brain, typically covering the basal portion of the skull and part of the neck (Figure 1a).^4,5^ With this method, all spins in the arteries within this volume will be inverted at once, and the perfusion measurement will typically be performed after a certain postlabel delay (*PLD*) or inversion time TI (Figure 1b). Finally, to control the exact timing of the labeling pulse, extra saturation volumes can be added after a time ${TI}_{1}$ .^6^ A second method involves repetitively applying a series of small volumes, forming a thin labelling plane of typically about 1 cm in thickness over a relatively long time, using reduced flip angles in a method dubbed pseudocontinuous arterial spin labeling or pCASL (Figure 1c & d).^7^ This method works by inverting the spins while they progress through these small volumes in a pseudo adiabatic fashion. It has several advantages over the PASL method, in that it allows for a much longer bolus of arterial labeling and ensures that all spins are labeled as close to the tissue of interest as possible, thereby theoretically increasing the SNR of the ASL sequence by a factor √2 .^3^ Finally, a third method has more recently been developed, based on a completely different labeling scheme, in which arterial water spins are labeled (either saturated or inverted) based on their velocities (Figure 1e & f). This method is therefore dubbed velocity-selective arterial spin labeling, or VSASL.^8^ The main advantage of this ASL sequence over the other two is that spins can be labeled much closer to the tissue of interest, thereby drastically reducing the arterial transit time. [Figure 1]

A consensus paper by the ISMRM Perfusion Study Group and European Consortium for ASL in Dementia^3^ describes in more detail all aspects of the first two techniques for both acquisition and processing steps to produce CBF maps. It also provides a clear set of parameters to be used to get a good first approximation of CBF measurements in multiple clinical conditions. Briefly, the measured difference image $∆M=M_{C}-M_{L}$ needs to be scaled by a proton-density map of the tissue $M_{0}$ and a series of other parameters to produce a CBF map using the following equations, depending on whether PASL or pCASL is used:

$CBF=6000\frac{\lambda }{2\alpha }\frac{\Delta M}{M_{0}}\frac{e^{\frac{TI}{T_{1b}}}}{{TI}_{1}}$ [mL/min/100g], for PASL [1]

$CBF=6000\frac{\lambda }{2\alpha }\frac{\Delta M}{M_{0}}\frac{e^{\frac{PLD}{T_{1b}}}}{T_{1b}\left(1-e^{-\frac{\tau }{T_{1b}}}\right)}$ [mL/min/100g], for PCASL [2]

In both equations, the factor 6000 allows to set the units in the historical values of mL per min per 100g of tissue. With volumes in both the numerator and denominator, the resulting units are in units of [1/Time], which is by definition a rate. In addition, both equations are very similar and differ only in the last part. The term $\frac{\lambda }{2\alpha }$ scales the blood flow by the blood : brain partition coefficient λ and the labeling efficiency α. The main data being scaled is the $\frac{\Delta M}{M_{0}}$ term, representing the difference between both images normalized by the proton density-weighted image $M_{0}$ . In both equations, $T_{1b}$ is the blood $T_{1}$ relaxation time, while *TI* and *TI*_1_ are defined in Figure 1b an *PLD* and *τ* in Figure 1d.

One of the main assumptions for these equations to be valid, and for images to truly reflect the amount of blood delivered to the brain, is that the PLD needs to be long enough for spins to be delivered via the microvasculature into the tissue.^9^ This is the greatest problem with simple single-delay ASL methods: while this assumption can be accepted in general for most clinical applications *without* vessel disease, it is often incorrect in subjects of young or old age and various slow-flow conditions, *e.g.* arterial occlusion or abnormal circulation.^10^ For that reason, it can be preferable to use multiple acquisitions at various PLDs to completely characterize the whole dynamics of the arterial blood bolus when it passes through the brain. This approach is referred to as multidelay ASL (MDASL), for which each MRI vendor has developed a slightly different labeling approach. Of note, VSASL is not typically used with multiple delays, as labeling is already performed very close to the tissue of interest throughout the brain.

### Labeling techniques

#### Look-Locker technique

**Figure 2.**
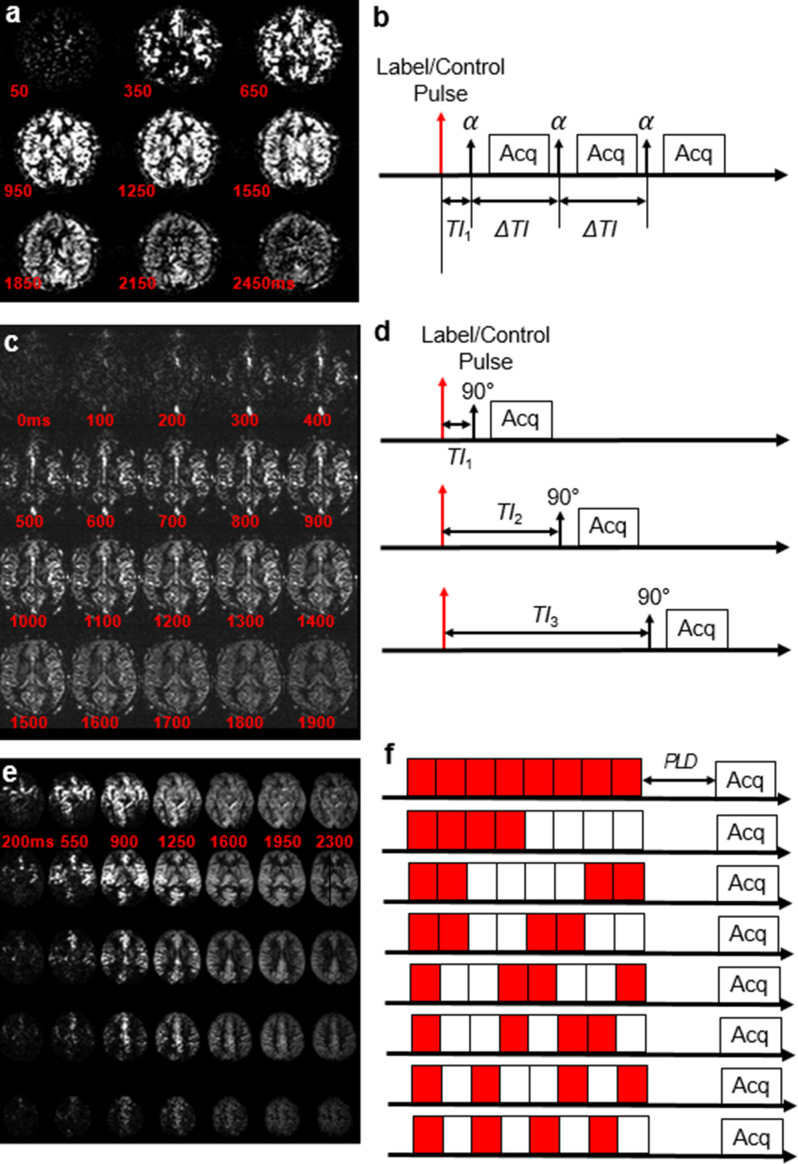
Multidelay ASL images acquired using the Look-Locker technique (**a**), Multi-TI technique (**c**), and Hadamard encoding (**e**). The pulse sequence timings are represented on the right hand side. For the Look-Locker technique (**b**), a single acquisition is needed to acquire all images, using a small flip angle α. For the Multi-TI technique, the same sequence is basically repeated multiple times after various inversion times *TI_n_*. Finally in the case of Hadamard encoding (here as an example using Walsh-ordered encoding steps^14^), for each acquisition, a different series of label (red) and control (white) steps is applied, and a numerical algorithm allows to reconstruct individual perfusion-weighted images at different Δ*TI*s. Figure 2c, courtesy of M. Guenther. Figure 2e adapted from^14^ with permission.

The Look-Locker encoding method was the first to be developed^11,12^ and is based on a simple PASL scheme, followed by a series of equally spaced acquisitions, using a small flip angle together with a multislice gradient echo EPI readout. Generally, this approach is relatively rapid as it allows for acquisition of multiple TI s in one acquisition, but presents with significant drawbacks compared to standard ASL sequences: 1) much lower SNR, due to a small flip angle readout to save magnetization until later time points; 2) SNR is further reduced by a readout that is typically multislice rather than 3D; 3) limited imaging volume, due to the trade-off between number of inversion times and coverage. A key advantage is complete kinetic characterization of the bolus, including bolus arrival time (BAT) to tissue, which enables accurate quantification.^13^ This method is currently provided by *Philips Healthcare* (Best, Netherlands). It usually requires up to 50 averages to provide satisfactory SNR, which with a typical TR of 3 s, results in a total scan time of 5 min. [Figure 2a–b]

#### Multi-TI

Another way of implementing MDASL is by acquiring several single-delay ASL images with either a PASL or pCASL labeling scheme.^15^ As 3D readouts are much more SNR-effective, this method does not require many repetitions for each TI .^16^ Potential disadvantages of this sequence are: 1) since each measurement is completely independent, there may be subject motion between different TI s; 2) generally, it takes up to 1 min per single TI to achieve adequate SNR, which forces a compromise between SNR and number of inversion times. This method also allows for quantitative assessment of the BAT.^15^ This method is implemented by *Siemens Healthineers* (Enrlangen, Germany) using either labeling and 3D-GRASE readout. [Figure 2c–d]

#### Hadamard encoding

A final approach to MDASL is Hadamard encoding,^14^ which takes advantage of the multiple acquisitions required for sufficient SNR to vary the labeling scheme during successive averages. Mathematical processing can then be used to synthesize post-hoc ASL images at various inversion times. Because of the improved SNR of this method over standard multi-TI,^14^ the entire sequence can usually be contained within a few minutes. This method seems to provide the best compromise to maximize both coverage and number of delays. However, the data require special pre-processing, which increases the risks of failure if any of the datasets is corrupted. This technique is implemented by *GE Healthcare* (Chicago, IL), with the bolus split into seven subboli of different temporal labelling and control sequences. A minimum of 8 averages is needed to reconstruct all images with varying PLDs. Each of the eight images can be added or subtracted in a combinatorial fashion to recreate ASL-weighted images at various PLDs. For example, the ASL-weighted image corresponding to bolus seven can be reconstructed from all data using the following equation:

[3] $$∆M\left(bolus7\right)=Im1-Im2-Im3+Im4-Im5+Im6+Im7-Im8$$

[Figure 2e–f]

### Quantitative metrics

While single-delay ASL permits assessment of CBF, MDASL enables calculation of several additional perfusion metrics. By following the bolus of labeled arterial blood through tissue, the arterial transit time (ATT) can be reported as the time in ms at which the signal ΔM appears in the tissue of interest. In single-delay ASL, perfusion-weighted signal can be absent or suboptimal depending on the choice of times *TI* or *PLD*. With MDASL, the acquisition of perfusion-weighted images at multiple time points enables calculation of an ATT-corrected CBF, in which some of the assumptions for Equations [1] and [2] are now fulfilled. Multiple methods can be utilized to fit the measured signal and calculate perfusion parameters using models^17,18^ or model-free methods,^13^ analogous to other clinical perfusion techniques such as dynamic susceptibility contrast (DSC).^19^ ASL uses blood water as an endogenous tracer, which distributes freely throughout the entire tissue intravascular and extravascular space. Perfusion-weighted signal comes from the arterial vasculature, primarily the distal vascular tree within small arterioles prior to reaching the capillary system. The majority of the labeled water is extracted on first passage, where it almost instantaneously exchanges with extravascular tissue water. More advanced models utilize arterial cerebral blood volume (ACBV) as a scaling factor in units of mL/100g.^13,17^ Estimation of total cerebral blood volume (CBV) requires an endogenous or exogenous tracer, such as the gadolinium-based contrast agents used in DSC, to quantify signal from the intravascular space.^19^

## Technical artefacts

MDASL considerably improves the accuracy and reproducibility of ASL in patient populations for which ATT is often unknown.^20–22^ However, the more basic models for CBF calculation do not correct for intravascular signal, which can lead to incorrect overestimation of perfusion values due to the large signal present in arteries.^10^ Many of the algorithms implemented on clinical platforms are indeed quite simple, such that the automatically generated perfusion values need to be assessed with caution. One of the main assumptions for the model equations to be valid, and for ASL images to truly reflect the amount of blood delivered to the brain, is that the PLD needs to be long enough for spins to arrive in the tissue of interest.^9^ This is the greatest problem with single-delay ASL methods: while an average PLD assumption is acceptable for many clinical applications *without* vascular disease, it is often incorrect in subjects who are very young or old, as well as for slow-flow disorders, *e.g.* arterial stenosis or occlusion.^10^ For that reason, it can be preferable to use multiple acquisitions at various PLDs to fully characterize the dynamic profile of the arterial blood bolus as it passes through the brain.

In MDASL, transit time correction weights CBF toward longer PLDs, and is therefore optimally utilized in conditions of slow flow.^23–27^ In young children, particularly newborns and infants, the overall flow velocity is slow and it becomes difficult to estimate at what time a bolus of arterial blood will enter the brain.^28^ Pathologic conditions such as stroke, vasculopathy, and hypoxic-ischemic injury can also decrease overall flow. The arterial transit artefact (ATA) is identified by the prolonged retention of spins within the arterial vasculature at PLDs when they should normally undergo capillary exchange.^29,30^ The use of longer labels makes the MDASL approach very important for accurate flow quantification in steno-occlusive disorders. In situations involving elevated flow, *e.g.* ictal epilepsy or infection, transit time correction may artifactually mask perfusion differences. This quantification error can be minimized by utilizing more complex models that also account for ACBV.^17^

As for any clinical sequence, there are practical tradeoffs between time and information. Different techniques for MDASL may result in longer imaging times, or conversely lower SNR for the same imaging time since measurements are split between different PLDs. Other artefacts, such as head positioning, motion, and susceptibility, are analogous to single-delay ASL and can be further complicated by differences between individual acquisitions.^30–32^ A number of protocol optimization techniques have been developed to reduce artefacts and improve contrast in MDASL.^33–41^

## Clinical applications

### Stroke

**Figure 3.**
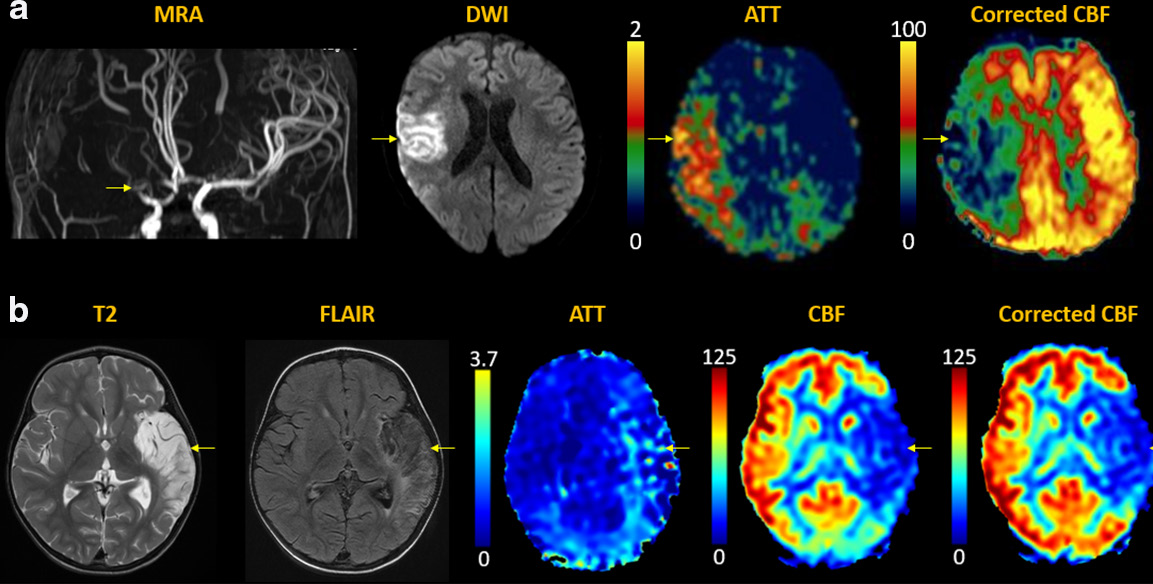
Arterial stroke. (**a**) Acute right MCA infarct with abrupt arterial cutoff and restricted diffusion (arrows). MDASL shows prolonged ATT with arterial transit artefact (arrows), indicating slow flow with attempted leptomeningeal collateralization. Transit time-corrected CBF is decreased (arrows) beyond the area of core infarct. This DWI-ASL mismatch indicates tissue at risk (ischemic penumbra), which can theoretically be rescued by early recanalization. In the absence of intervention, this region is likely to progress to completed infarct. (**b**) Subacute left MCA infarct, with edema evolving to encephalomalacia (arrows). MDASL shows minimal attempted collateralization with arterial transit artefact on ATT map and matched decrease in CBF (arrows). Transit time correction improves estimation of normal CBF and homogeneity across the field-of-view.

Acute stroke can be arterial or venous in aetiology. Arterial infarcts are caused by stenosis or occlusion of arteries supplying brain tissue, reducing cerebral blood flow and leading to energy failure with cytotoxic parenchymal injury. The severity of cerebral ischemia has been linked to ATA and CBF measurements on ASL,^42,43^ and accuracy improves with multidelay approaches.^44–47^ MDASL metrics have also been correlated with other perfusion imaging modalities including DSC MRI, computed tomography (CT) perfusion, single photon emission computed tomography (SPECT), and positron emission tomography (PET).^48–50^ [Figure 3]

**Figure 4.**
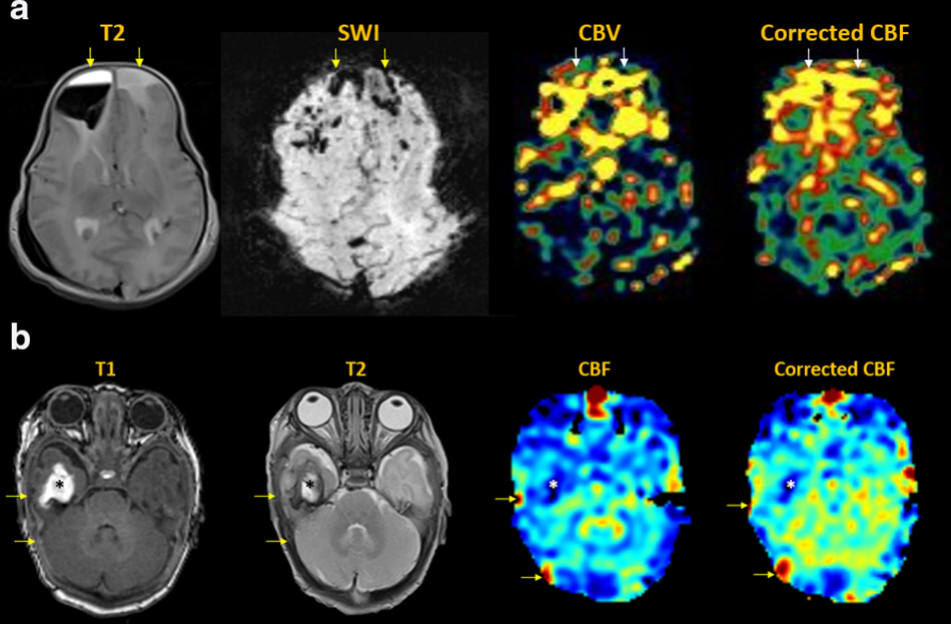
Venous stroke. (**a**) Acute venous infarct in patient with genetic malformations and coagulopathy. Bifrontal hematomas with layering fluid-blood levels, cortical and medullary venous thrombosis (arrows). MDASL with transit time correction shows elevated bifrontal ACBV and CBF, reflecting combined venous congestion and inflammation. (**b**) Chronic right anterior temporal hemorrhagic venous infarct (black asterisks) with thrombus in the right vein of Labbe and transverse sinus (arrows). MDASL shows decreased flow to the right anterior temporal lobe (white asterisks) with high venous signal in the transverse sinus (arrows). Transit time correction better quantifies flow to parenchyma.

Venous infarcts result from thrombosis within the deep and/or superficial draining cerebral veins, including the dural venous sinuses. This results in outflow obstruction with blood-brain barrier disruption and vasogenic edema. Over time, rising tissue pressures can lead to parenchymal hemorrhage and secondary arterialization of stroke due to inflow impairment. On ASL, the “bright sinus” sign is a harbinger of trapped arterial spins proximal to the venous obstruction and can resolve following antithrombotic therapy.^51–53^ MDASL helps to more accurately quantify perfusion metrics in the setting of slow flow. [Figure 4]

### Vasculopathy

**Figure 5.**
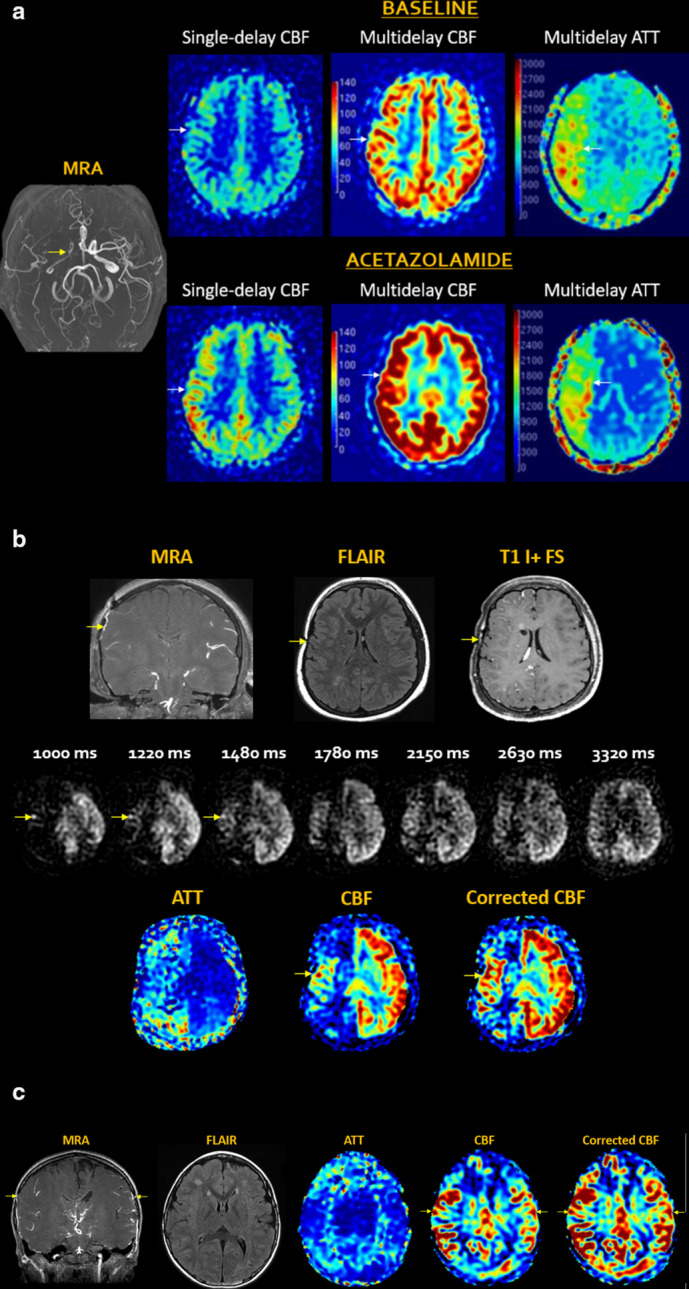
Moyamoya disease. (**a**) Right moyamoya disease with high-grade stenosis of the right carotid terminus and branches (yellow arrow), with incomplete reconstitution of distal branches via leptomeningeal collaterals. In this slow-flow condition, single-delay ASL markedly underestimates CBF. MDASL more accurately calculates CBF by accounting for slow flow through collateral vessels at long PLDs. At rest, there is increased ATT and decreased CBF in the right MCA distribution (arrows). Following intravenous acetazolamide challenge, maximal vasodilation induces diffusely elevated perfusion with reduction of ATT and CBF defects. This indicates intact residual cerebrovascular reserve, meaning that the patient is not at immediate risk of ischemia, and surgery can be delayed. (**a**) Down syndrome with right moyamoya post pial synangiosis (arrows). Chronic white matter ischemia and lacunar infarcts are present in the right hemisphere, with overlying enhancing leptomeningeal collaterals. seven consecutive PLDs demonstrate slow retrograde flow through the synangiosis (arrows) into the right MCA territory. There is progressive regional improvement in right hemispheric perfusion defects, with residual borderzone hypoperfusion. MDASL shows persistently increased ATT and decreased CBF to the right external vascular borderzones, better quantified after transit time correction. (**b**) Bilateral moyamoya disease with high-grade ICA occlusions, post bilateral synangiosis (arrows) communicating with leptomeningeal vessels. Multifocal lacunar infarcts are present, with numerous lenticulostriate and thalamoperforator moyamoya collaterals. MDASL shows patent synangioses with decreased ATT and increased CBF (arrows). Slow flow through synangiosis and collateral vessels is reflected in transit time-corrected CBF. There is residual hypoperfusion to the external vascular borderzones with elevated ATT and decreased CBF.

Moyamoya disease is an obliterative vasculopathy that causes progressive stenosis of cerebral vessels, most commonly the supraclinoid internal carotid arteries. When associated with a predisposing condition, such as neurofibromatosis, Down syndrome, sickle cell anaemia, or radiation, it is known as moyamoya syndrome. Compensatory formation of multiple tortuous collateral vessels in the lenticulostriate, basal, and leptomeningeal circulations produces the characteristic “puff-of-smoke” appearance and “ivy sign” on angiography. By far, the most well-established application of MDASL is for quantifying perfusion in moyamoya patients, since flow through collateral vessels is far slower and more disorganized than in the normal arterial tree. As a result, short-delay ASL performed at conventional PLDs can yield a false-positive impression of ischemia. Longer labels are required to more accurately characterize the delayed inflow to brain parenchyma through collateral pathways.^54,55^ In both preoperative and postoperative moyamoya patients, MDASL has been used to effectively characterize the degree of collaterals, tissue oxygenation, and cerebrovascular reserve (CVR) using acetazolamide or other vasoactive challenges.^56–58^ These metrics have also been benchmarked against other perfusion imaging modalities.^59–63^ [Figure 5]

**Figure 6.**
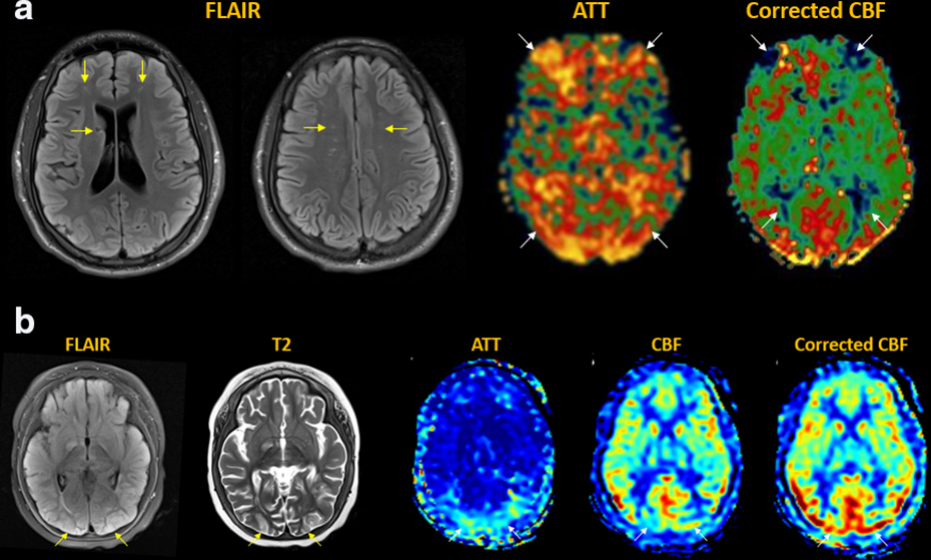
Other vasculopathies. (**a**) Sickle cell anaemia with scattered lacunar infarcts and radiating linear white matter FLAIR hyperintensities (yellow arrows). MDASL with transit time correction shows increased ATT and decreased CBF to the bilateral vascular watershed zones, between major arterial territories and within deep white matter (white arrows). (**b**) Posterior reversible encephalopathy with edema in the deep gray nuclei, parieto-occipital cortex and white matter (yellow arrows). MDASL shows increased ATT and decreased CBF to the bilateral posterior watershed zones with arterial transit artefact (white arrows). Transit time correction increases sensitivity for seizure-induced cortical hyperperfusion, with the watershed hypoperfusion areas less apparent.

Flow dynamics of other vasculopathies affecting the large, medium, and small vessels can also be characterized using MDASL. In comparison with the adult population, where such cases are often attributed to atherosclerosis or drug use, pediatric vasculopathies tend to be milder and in occasionally reversible. Major etiologies include connective tissue disorders, blood dyscrasias, inflammatory/infectious conditions, and vascular dysautoregulation.^64–72^ [Figure 6]

### Hypoxic-Ischemic injury

Hypoxic-ischemic injury (HII) has different imaging manifestations, depending on the severity and duration of cerebral flow compromise, as well as the timing of imaging after injury. Age of is also very important: children experience higher flow to actively developing brain regions, thus makes these areas selectively vulnerable in the setting of profound HII.^73^ In cases of mild or partial HII, overall decreased cerebral inflow results in watershed infarcts at the junctions of cerebral artery territories. On ASL, this manifests as the “borderzone” sign with symmetrically symmetric wedge-shaped areas of hypoperfusion along the anterior/middle and middle/posterior cerebral artery borderzones.^74,75^ With severe and prolonged HII, critical and highly metabolic structures are affected including the cerebral cortex, basal ganglia, hippocampi, and cerebellum. Global anoxic injury may occur in the setting of cardiac arrest, attempted hanging, motor vehicle accidents, abusive head injury, and iatrogenic etiologies.^76–79^

**Figure 7.**
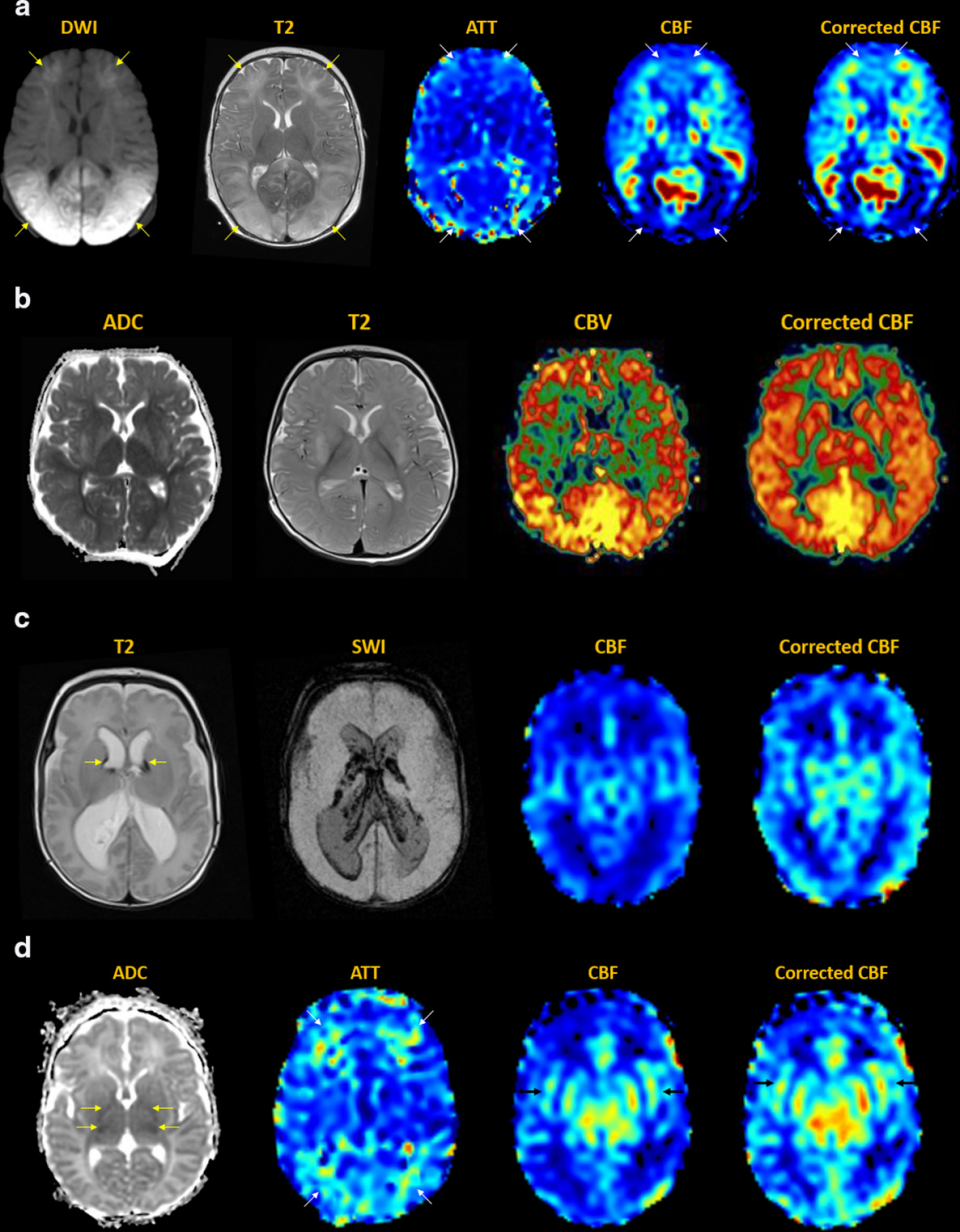
Hypoxic-ischemic injury.(**a**) Watershed infarcts in patient with coagulopathy and septic shock. DWI shows bilateral external watershed infarcts (yellow arrows). MDASL shows mildly elevated ATT and decreased CBF in the external vascular borderzones (white arrows). (**b**) Acute anoxic injury post-cardiac arrest with diffuse cerebral edema and diffusion restriction noted throughout gray and white matter. MDASL with transit time correction shows prominent rebound hyperperfusion with diffusely elevated CBF and ACBV. (**c**) Preterm birth injury with Grade 3 intraventricular hemorrhage involving the bilateral caudothalamic grooves (arrows), choroid plexi, and ventricular ependyma with hydrocephalus. There is immature sulcation and patchy white matter injury with faint periventricular T2 hyperintense signal. MDASL shows decreased periventricular white matter perfusion, with more accurate estimation following transit time correction. (**a**) Term birth injury. ADC map shows mild diffusion restriction in the posterior limbs of internal capsules and ventrolateral thalami (yellow arrows), as well as T2 hyperintense white matter signal in the external vascular borderzones. MDASL shows elevated ATT to the external vascular borderzones (white arrows). CBF shows mild rebound hyperperfusion to the bilateral basal ganglia and corticospinal tracts (black arrows), representing areas of perinatal selective vulnerability. Transit time correction improves estimation of brain perfusion. (**b**) Neonatal abstinence syndrome in an infant born to an opioid-dependent mother. MDASL is useful in accurately quantifying neonatal brain perfusion, which is typically low at birth. Increased CBF at birth has been observed both globally and regionally in NAS babies relative to normal controls. Opioids alter CBF depending on baseline cerebrovascular tone, such that acute drug withdrawal likely impacts both cerebral autoregulation and autonomic activity.

MDASL is highly useful in assessing newborns and infants, who have low CBF values at baseline. Overall flow velocities may be further reduced in the setting of perinatal asphyxia and other birth complications.^28,80^ In preterm infants, the cerebral circulation and neuroglial progenitor cells are immature, such that ischemic injury impacts the draining medullary veins and germinal matrix remnants. This can lead to white matter injury and intraventricular hemorrhage, respectively. In term infants, the actively developing corticospinal tracts and basal ganglia are selectively vulnerable.^81–84^ Therapeutic hypothermia is the clinical standard for neuroprotection, and is intended to minimize rebound hyperperfusion leading to secondary energy failure with brain necrosis. ASL is a useful biomarker for quantifying hyperperfusion injury to vulnerable structures, and the resulting ischemic steal from other areas of brain.^84^ Neonatal abstinence syndrome refers to infants with *in utero* opioid exposure from maternal drug use. At birth, infants suffer from opioid withdrawal autonomic, motor, and sensory dysregulation. MDASL perfusion metrics are elevated both globally and regionally, and correlate with abnormalities on neurologic examination.^85^ [Figure 7]

### Epilepsy

Epilepsy is a complex disorder characterized by recurrent unprovoked seizures with synchronized neuronal hyperexcitability. The International League Against Epilepsy has established a framework for classification of the epilepsies. Seizures can be focal in onset, with localizing neurologic symptoms; or generalized with diffuse involvement of the brain. Etiologies are diverse and include structural, vascular, infectious, genetic, metabolic, and immune causes.^86–88^ In the pediatric population, congenital brain malformations are an important diagnostic consideration. Focal cortical dysplasia (FCD) is a cause of medically refractory focal epilepsy in children that is challenging yet important to diagnose early, as appropriate intervention may enable seizure-free outcomes.^89^

**Figure 8.**
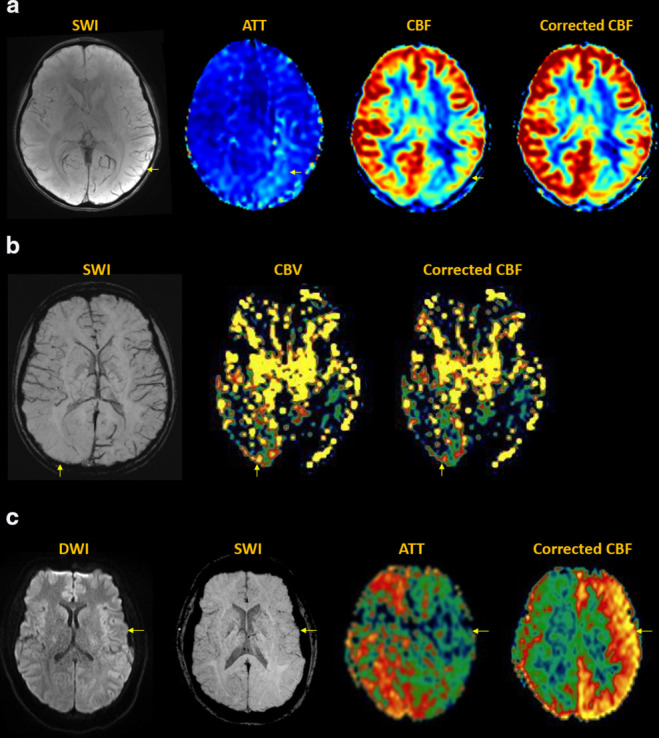
Epilepsy. (**a**) Cortical dysplasia of the right parieto-occipital region, showing disorganized sulcation with irregular gray-white junction (dotted circle). MDASL performed in interictal phase demonstrates corresponding focally decreased flow to the area of dysplasia (arrows). Because seizures are a high-flow phenomenon, transit time correction does not significantly alter the CBF results in normal brain. The area of hypoperfusion is present, though less apparent due to weighting towards longer PLDs. (**b**) Sturge-Weber of the left posterior quadrant with enhancing dysplastic veins in the subarachnoid space, parenchymal atrophy and gyriform calcifications (arrows). MDASL shows increased ATT and decreased CBF in the affected region (arrows). Transit time correction leads to better and more homogeneous flow quantification in normal brain. (**c**) Infantile spasms in West syndrome. Anatomic imaging is normal. MDASL shows diffusely elevated perfusion to cerebral cortex and basal ganglia. Findings are similar after transit time correction in this high-flow condition. (**d**) Chronic epilepsy in patient with Down syndrome. There is mild global volume loss and white matter signal abnormality. MDASL shows prolonged ATT and decreased CBF throughout the brain, more so along the external vascular borderzones. Transit time correction improves estimation of brain perfusion.

Diagnostic evaluation of epilepsy consists of clinical, electrophysiologic, and imaging workup to identify the epileptogenic zone (EZ) responsible for seizure generation, which if surgically targeted leads to seizure freedom. Multimodal imaging approaches are used to lateralize and/or localize the EZ, including anatomic imaging and some combination of ASL, DSC, SPECT, and PET.^90–93^ Concordance between modalities increases the likelihood of seizure-free surgical outcomes. Perfusion imaging findings in epilepsy depend on seizure pattern and timing: in focal epilepsy, the seizure focus shows hypoperfusion in the interictal period and hyperperfusion in the peri-ictal period, with statistical subtraction increasing sensitivity for EZ localization.^93^ If imaging is performed after seizure onset, spreading hyperperfusion can be seen throughout the ipsilateral cerebral hemisphere. Associated limbic system connectivity can manifest with CBF increases in the ipsilateral hippocampus and thalamus (Papez circuit) and contralateral cerebellum (corticopontocerebellar pathway).^94^ In patients with status epilepticus, ongoing seizure activity may yield lead to diffuse cerebral hyperperfusion with excitotoxic complications.^95,96^ In patients with chronic epilepsy, MDASL can be helpful for longitudinal CBF quantification to assess treatment response (both medical and surgical). [Figure 8]

### Migraine

**Figure 9.**
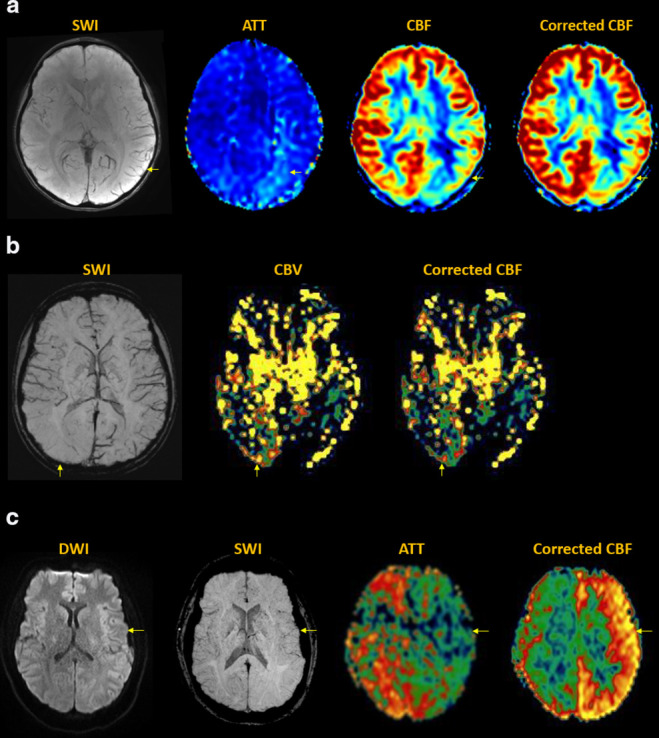
Migraine. (**a**) Migraine aura. Transient right facial droop and receptive (Wernicke) aphasia. MDASL shows elevated ATT and decreased CBF to the left motor strip and posterior quadrant in a non-arterial distribution, with corresponding cortical venous engorgement (arrows). Transit time correction improves estimation of overall brain perfusion, though the hypoperfused areas are less apparent. (**b**) Migraine cephalalgia. Several days of right headache with left homonymous hemianopsia. MDASL with transit time correction shows rebound hyperperfusion with elevated CBF and ACBV to the right posterior quadrant, including visual cortex (arrows). There is also decreased deoxyhemoglobin content within cortical veins on SWI. (**c**) Therapy-resistant right hemiplegic migraine. Subtle fullness and restricted diffusion of the left cerebral cortex, with decreased cortical venous susceptibility (arrows). MDASL shows rebound hyperperfusion with decreased ATT And increased CBF throughout the left cerebral hemisphere (arrows).

Migraine is a neurological condition characterized by recurrent headaches and associated symptoms that can include nausea, vomiting, photophobia, phonophobia, aphasia, vision changes, and weakness or paresthesias. Migraine presents in distinct phases: prodrome (premonitory phase); aura (focal neurological deficits); headache (cephalalgic phase); and postdrome (recovery phase). There is a familial and genetic association, with hemiplegic migraine linked to mutations in ion channel and transport proteins. The theorized pathogenesis relates to cortical spreading depression with cascading effects on neural and vascular function. In patients with migraine aura, there is characteristic decreased perfusion on ASL, with accompanying vasoconstriction in some cases. Patients experience transient stroke-like symptoms corresponding to the affected brain territory. Neurologic symptoms subsequently resolve in the cephalalgic phase, with rebound vasodilation leading to headache symptoms and elevated perfusion on ASL.^97–100^ Triptans are a family of serotonin receptor agonists used as abortive treatments for migraine. Therapeutic effectiveness can be monitored by ASL.^101^ [Figure 9]

### Trauma

**Figure 10.**
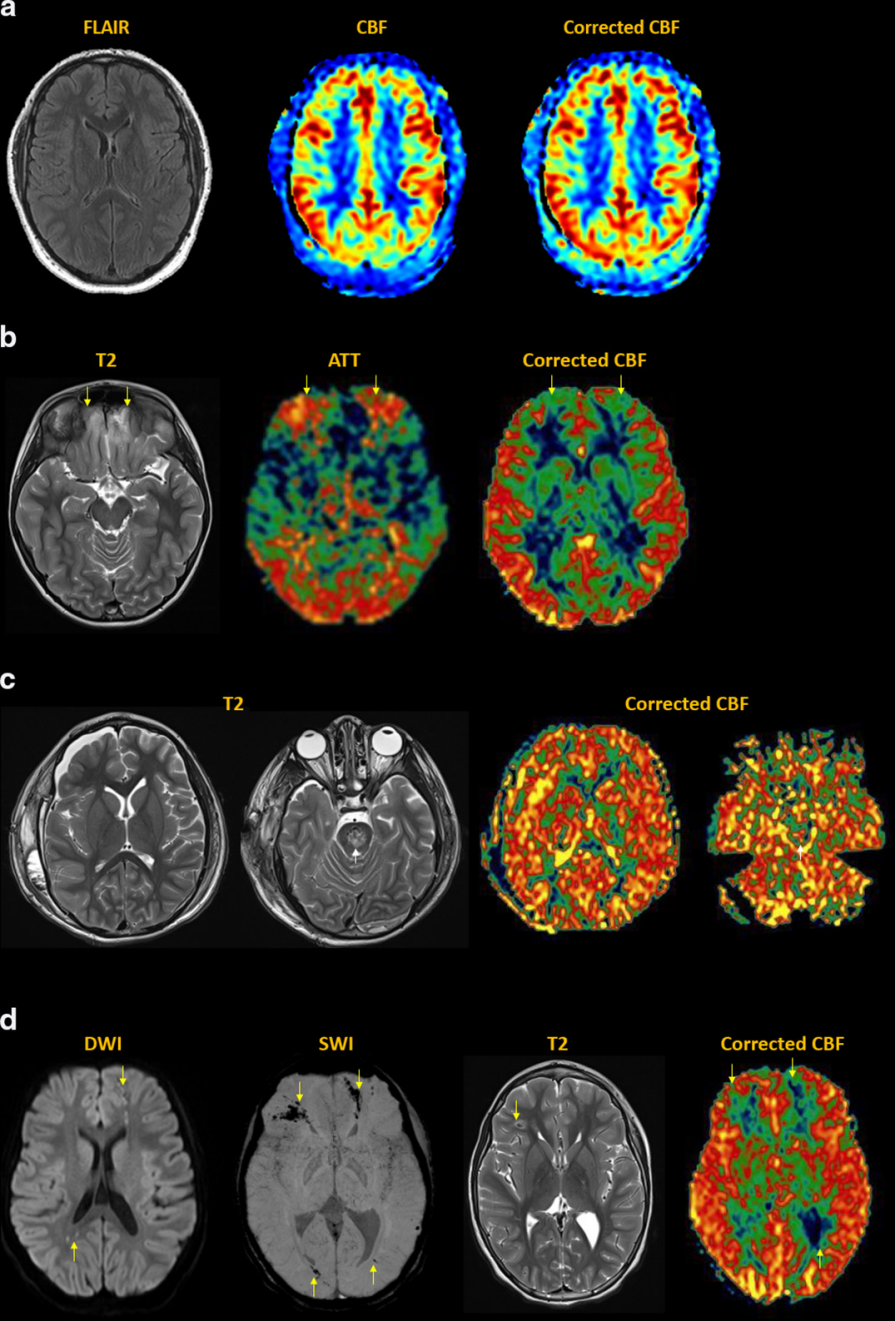
Trauma. (**a**) Concussion in high school athlete with persistent language difficulties. Anatomic MRI is normal. MDASL shows heterogeneous perfusion to gray and white matter, better quantified after transit time correction. (**b**) Low-impact trauma from fall with bifrontal hemorrhagic cerebral contusions (arrows). MDASL shows increased ATT and decreased corrected CBF (arrows). (**c**) Diffuse axonal injury from motor vehicle accident with comminuted fractures, subgaleal and extraaxial hemorrhage, pontine hematoma (arrows), and diffuse cerebral edema. MDASL shows heterogeneously elevated cerebral perfusion adjacent to hemorrhage, and hypoperfusion in areas of edema. (**d**) Diffuse axonal injury from motor vehicle accident. Microstructural shear injury involves the gray-white junction and deep white matter (arrows). MDASL shows heterogeneously reactive cortical flow and hypoperfusion to areas of injury.

Traumatic brain injury (TBI) has varying clinical manifestations depending on the mechanism, severity, and duration of injury. Anatomic imaging can be negative in mild TBI, though ASL may reveal perfusion abnormalities suggestive of occult cerebrovascular dysregulation. Low-impact injuries are associated with cerebral contusions, which tend to be greatest along the inferior frontal and anterior temporal lobes, adjacent to the rigid falx cerebri and sphenoid wings. In high-speed motor vehicle accidents, acceleration-deceleration injuries can yield diffuse axonal injury with shearing of the gray-white matter junction and deep structures. MDASL is helpful for quantifying cerebrovascular derangements in addition to the macrostructural findings. Multimodal advanced imaging approaches can help provide imaging biomarkers that better predict patient outcomes in TBI.^101^ Abusive head trauma can also be associated with anatomic and perfusion deficits, commonly related to head-shaking and/or strangulation mechanisms.^78^ [Figure 10]

### Tumor

**Figure 11.**
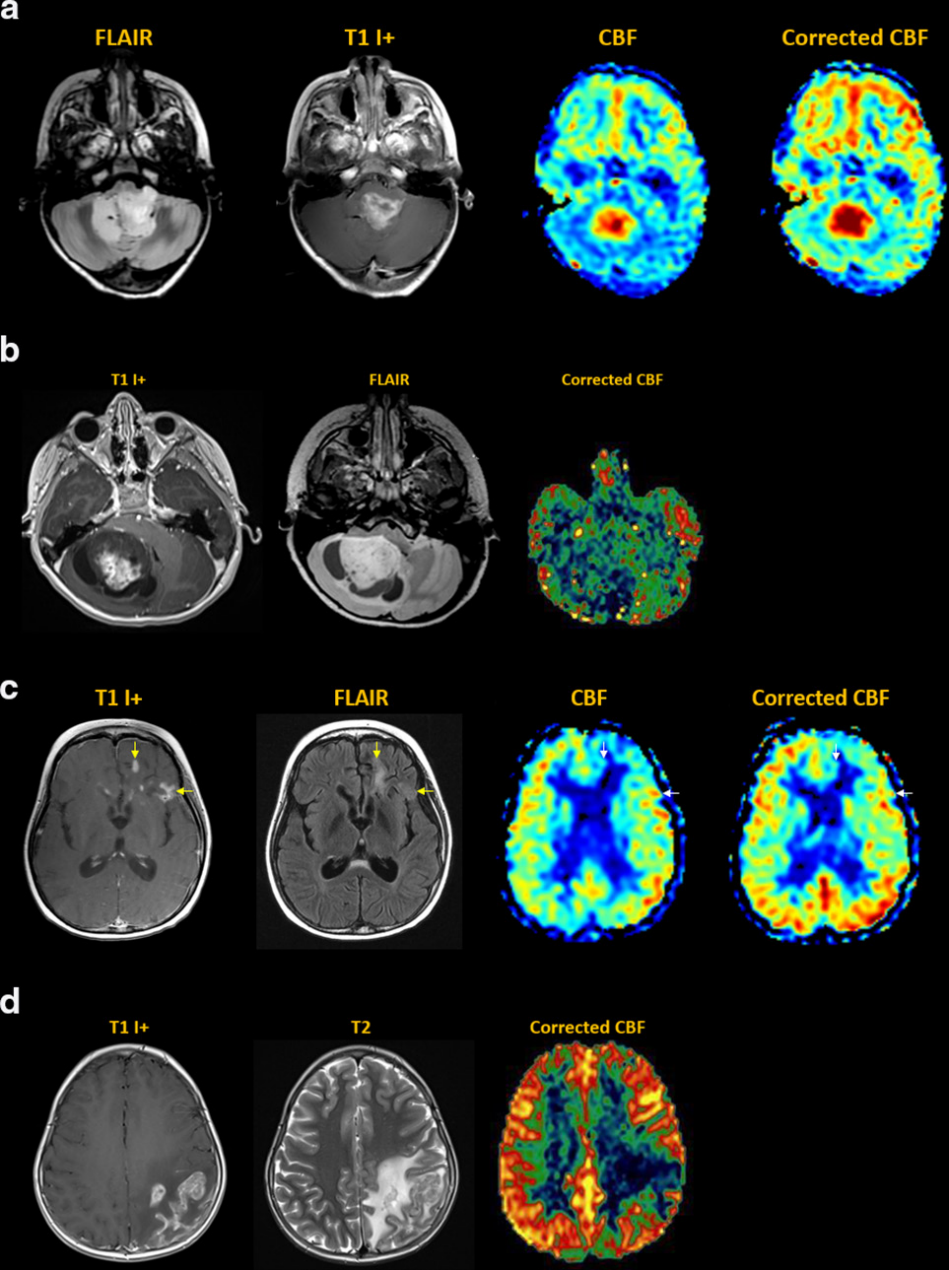
Tumor. (**a**) Ependymoma of fourth ventricle and left foramen of Luschka, with heterogeneous internal enhancement. MDASL shows elevated CBF within tumor, more accurately quantified following transit time correction. (**b**) Right cerebellar pilocytic astrocytoma with solid & cystic components. MDASL shows mildly elevated CBF within solid tumor, and decreased CBF within cystic components. (**c**) Recurrent disseminated medulloblastoma with left frontal lobe parenchymal and leptomeningeal metastases, demonstrating enhancement and surrounding vasogenic edema (yellow arrows). MDASL shows elevated perfusion within the metastases, on a background of posttreatment encephalomalacia. Tumoral flow is better detected after transit time correction (white arrows).(**d**) Metastatic rhabdomyosarcoma with infiltrative cortical enhancement and vasogenic edema of left parietal lobe. MDASL shows elevated perfusion within the metastasis, and decreased perfusion in the areas of edema.

Although DSC is the clinical standard for tumor perfusion evaluation, the use of intravenous gadolinium contrast is contraindicated in certain situations—*e.g.* renal failure with risk of nephrogenic systemic fibrosis, pregnant patients in whom contrast crosses the placental barrier. Especially in children, there are concerns regarding gadolinium tissue deposition, especially when the clinical indication merits multiple follow-up MRI examinations. The use of MDASL provides quantitative perfusion metrics for CBF and ACBV, which correlates with tumor histology, grade, and and histopathologic vascular density in multiple studies.^102–105^ ASL metrics also correlate well with DSC and other perfusion imaging modalities.^106^ [Figure 11]

### Infection/Inflammation

**Figure 12.**
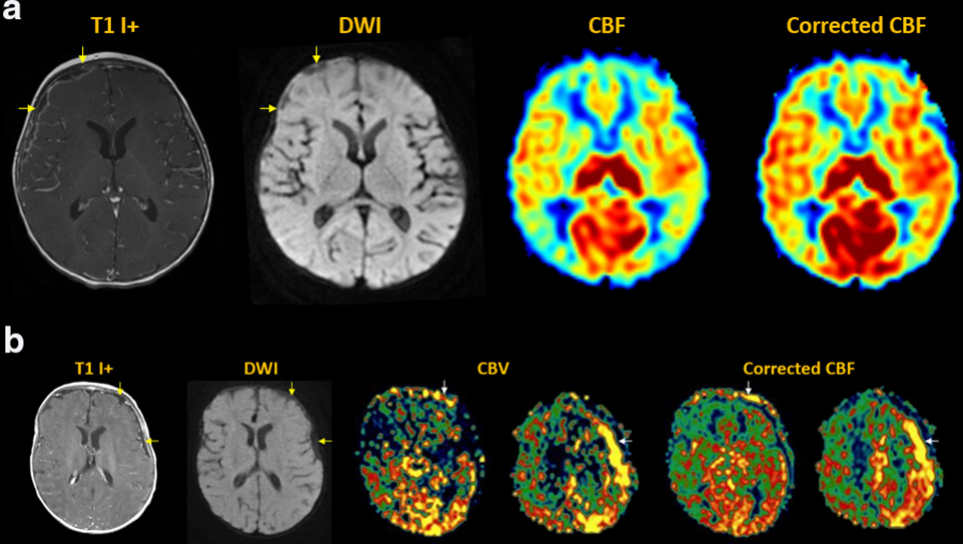
Infection. (**a**) Streptococcus pneumonia meningitis with leptomeningeal enhancement and right frontal subdural empyema (arrows). MDASL shows diffusely elevated cortical perfusion accompanying the meningeal inflammation, better estimated after transit time correction. (**b**) Group B streptococcus meningitis. Mild leptomeningeal enhancement with left subdural empyemas (yellow arrows). MDASL shows heterogeneously elevated flow (CBF and ACBV) along the left dura mater and cerebral cortex (white arrows).

Central nervous system infection can be caused by viral, bacterial, fungal, and parasitic organisms. Potential complications include meningitis, cerebritis, brain abscess, septic thrombophlebitis, and mycotic aneurysm. ASL is helpful in assessing areas of hyperemia or ischemia due to direct parenchymal or vascular involvement.^107,108^ Inflammatory and demyelinating conditions can also disrupt cerebral blood flow, presenting with hyperemia in acute disease, CBF normalization in the subacute stage, and low flow in chronic lesions.^109^ [Figure 12]

### Metabolic

**Figure 13.**
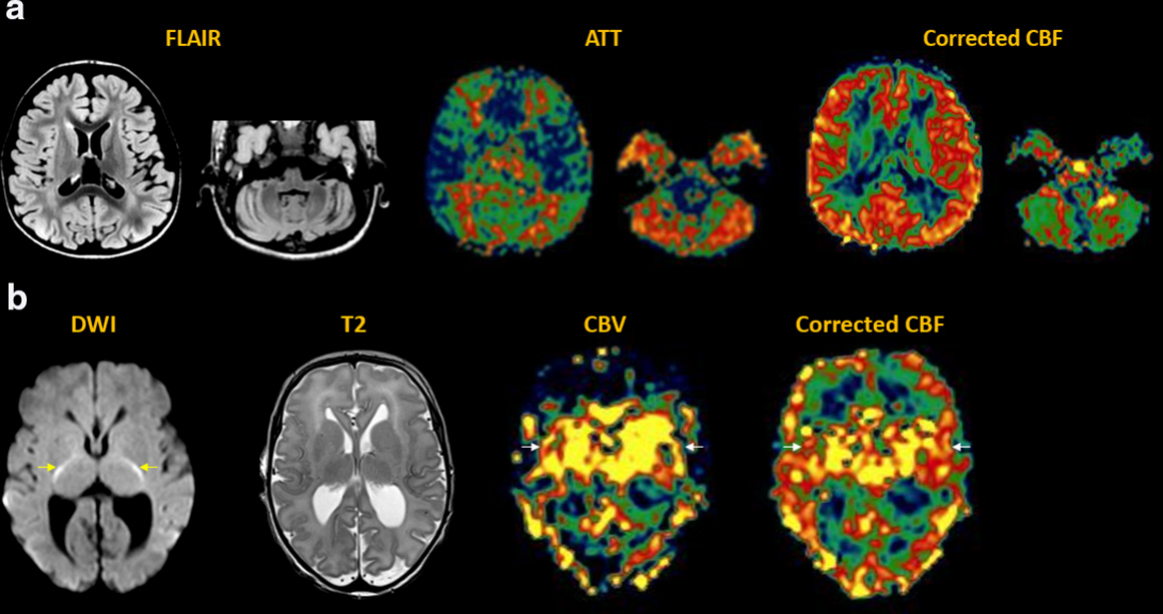
Metabolic disease. (**a**) Batten disease with patchy white matter signal abnormality, global cerebral and cerebellar volume loss. MDASL shows prolonged ATT in the external vascular borderzones, with heterogeneous decrease in white matter CBF. (**b**) Nonketotic hyperglycinemia with diffuse white matter edema and restricted diffusion in bilateral corticospinal tracts (yellow arrows). MDASL with transit time correction shows elevated ACBV and CBF in the basal ganglia and corticospinal tracts (white arrows), suggesting primary energy failure. There is low perfusion to the edematous white matter.

Inherited metabolic disorders are genetic conditions that impair normal energy metabolism. Hundreds of different mechanisms exist, including failure of energy production or utilization, accumulation of toxic intermediary metabolites, and challenges with complex molecule processing and transport. Depending on the affected gene(s) and mutational severity, the clinical presentation can vary widely in terms of age and timing of onset, symptoms, and organ systems affected. Episodes of illness or stress can precipitate or exacerbate energy decompensation.^110^ ASL can be useful for distinguishing metabolic stroke-like episodes from vascular stroke. In general, metabolic crises present with one or more areas of hyperperfusion in non-vascular territories., thought to represent transient energy failure with vascular reactivity and blood-brain barrier breakdown. Perfusion abnormalities can be seen in the preclinical stage of disease and may help predict cognitive deficits.^111–115^ [Figure 13]

## Conclusions

MDASL is a clinically robust technique for noncontrast evaluation of cerebral blood flow. MRI labeling approaches vary by vendor and include Look-Locker, multi-TI, and Hadamard encoding approaches. Compared to single-delay ASL, MDASL enables more accurate and reproducible quantification of CBF and other perfusion parameters, such as ATT and ACBV. Due to practical limitations in imaging time and SNR, as well as potential overestimation with simpler models, MDASL is most helpful in young subjects and slow-flow conditions. We review major clinical applications in the pediatric brain including stroke, vasculopathy, hypoxic-ischemic injury, epilepsy, migraine, tumor, infection, and metabolic disease.

## References

[1] Detre JA Leigh JS Williams DS Koretsky AP . Perfusion imaging . *Magn Reson Med* 1992 ; 23: 37 – 45 . doi: 10.1002/mrm.1910230106 1734182

[2] Williams DS Detre JA Leigh JS Koretsky AP . Magnetic resonance imaging of perfusion using spin inversion of arterial water . [ Internet ]. *Proc Natl Acad Sci U S A* 1992 ; 89: 212 – 16 . doi: 10.1073/pnas.89.1.212 PMC48206 1729691

[3] Alsop DC Detre JA Golay X Günther M Hendrikse J Hernandez-Garcia L et al . Recommended implementation of arterial spin-labeled perfusion MRI for clinical applications: A consensus of the ISMRM perfusion study group and the european consortium for ASL in dementia . *Magn Reson Med* 2015 ; 73: 102 – 16 . doi: 10.1002/mrm.25197 24715426 PMC4190138

[4] Kim SG . Quantification of relative cerebral blood flow change by flow-sensitive alternating inversion recovery (FAIR) technique: application to functional mapping . *Magn Reson Med* 1995 ; 34: 293 – 301 . doi: 10.1002/mrm.1910340303 7500865

[5] Edelman RR Chen Q . EPISTAR MRI: multislice mapping of cerebral blood flow . *Magn Reson Med* 1998 ; 40: 800 – 805 . doi: 10.1002/mrm.1910400603 9840822

[6] Wong EC Buxton RB Frank LR . Quantitative imaging of perfusion using a single subtraction (QUIPSS and QUIPSS ii) . *Magn Reson Med* 1998 ; 39: 702 – 8 . doi: 10.1002/mrm.1910390506 9581600

[7] Dai W Garcia D BC Alsop DC . Continuous flow-driven inversion for arterial spin labeling using pulsed radio frequency and gradient fields . *Magn Reson Med* 2008 ; 60: 1488 – 97 . doi: 10.1002/mrm.21790 19025913 PMC2750002

[8] Wong EC Cronin M Wu WC Inglis B Frank LR Liu TT . Velocity-selective arterial spin labeling . *Magn Reson Med* 2006; 55: 1334–41. 10.1002/mrm.20906 16700025

[9] Alsop DC Detre JA . Reduced transit-time sensitivity in noninvasive magnetic resonance imaging of human cerebral blood flow . *J Cereb Blood Flow Metab* 1996 ; 16: 1236 – 49 . doi: 10.1097/00004647-199611000-00019 8898697

[10] Bokkers RPH van der Worp HB Mali WPTM Hendrikse J . Noninvasive MR imaging of cerebral perfusion in patients with a carotid artery stenosis . *Neurology* 2009 ; 73: 869 – 75 . doi: 10.1212/WNL.0b013e3181b7840c 19752454

[11] Hendrikse J Lu H van der Grond J Van Zijl PCM Golay X . Measurements of cerebral perfusion and arterial hemodynamics during visual stimulation using turbo-TILT . *Magn Reson Med* 2003 ; 50: 429 – 33 . doi: 10.1002/mrm.10525 12876722

[12] Günther M Bock M Schad LR . Arterial spin labeling in combination with a look-locker sampling strategy: inflow turbo-sampling EPI-FAIR (ITS-FAIR) . *Magn Reson Med* 2001 ; 46: 974 – 84 . doi: 10.1002/mrm.1284 11675650

[13] Petersen ET Lim T Golay X . Model-free arterial spin labeling quantification approach for perfusion MRI . *Magn Reson Med* 2006 ; 55: 219 – 32 . doi: 10.1002/mrm.20784 16416430

[14] von Samson-Himmelstjerna F Madai VI Sobesky J Guenther M . Walsh-ordered hadamard time-encoded pseudocontinuous ASL (WH pcasl) . *Magn Reson Med* 2016 ; 76: 1814 – 24 . doi: 10.1002/mrm.26078 26714671

[15] MacIntosh BJ Filippini N Chappell MA Woolrich MW Mackay CE Jezzard P . Assessment of arterial arrival times derived from multiple inversion time pulsed arterial spin labeling MRI . *Magn Reson Med* 2010 ; 63: 641 – 47 . doi: 10.1002/mrm.22256 20146233

[16] Günther M Oshio K Feinberg DA . Single-shot 3D imaging techniques improve arterial spin labeling perfusion measurements . *Magn Reson Med* 2005 ; 54: 491 – 98 . doi: 10.1002/mrm.20580 16032686

[17] Chappell MA MacIntosh BJ Donahue MJ Günther M Jezzard P Woolrich MW . Separation of macrovascular signal in multi-inversion time arterial spin labelling MRI . *Magn Reson Med* 2010 ; 63: 1357 – 65 . doi: 10.1002/mrm.22320 20432306

[18] Buxton RB Frank LR Wong EC Siewert B Warach S Edelman RR . A general kinetic model for quantitative perfusion imaging with arterial spin labeling . *Magn Reson Med* 1998 ; 40: 383 – 96 . doi: 10.1002/mrm.1910400308 9727941

[19] Østergaard L . Principles of cerebral perfusion imaging by bolus tracking . *J Magn Reson Imaging* 2005 ; 22: 710 – 17 . doi: 10.1002/jmri.20460 16261573

[20] Guo J Holdsworth SJ Fan AP Lebel MR Zun Z Shankaranarayanan A et al . Comparing accuracy and reproducibility of sequential and hadamard-encoded multidelay pseudocontinuous arterial spin labeling for measuring cerebral blood flow and arterial transit time in healthy subjects: A simulation and in vivo study . *J Magn Reson Imaging* 2018 ; 47: 1119 – 32 . doi: 10.1002/jmri.25834 28792653 PMC5807238

[21] Gevers S van Osch MJ Bokkers RPH Kies DA Teeuwisse WM Majoie CB et al . Intra- and multicenter reproducibility of pulsed, continuous and pseudo-continuous arterial spin labeling methods for measuring cerebral perfusion . *J Cereb Blood Flow Metab* 2011 ; 31: 1706 – 15 . doi: 10.1038/jcbfm.2011.10 21304555 PMC3170937

[22] Lin T Qu J Zuo Z Fan X You H Feng F . Test-retest reliability and reproducibility of long-label pseudo-continuous arterial spin labeling . *Magn Reson Imaging* 2020 ; 73: 111 – 17 : S0730-725X(20)30092-8 . doi: 10.1016/j.mri.2020.07.010 32717203

[23] Qin Q Huang AJ Hua J Desmond JE Stevens RD van Zijl PCM . Three-dimensional whole-brain perfusion quantification using pseudo-continuous arterial spin labeling MRI at multiple post-labeling delays: accounting for both arterial transit time and impulse response function . *NMR Biomed* 2014 ; 27: 116 – 28 . doi: 10.1002/nbm.3040 24307572 PMC3947417

[24] Martin SZ Madai VI von Samson-Himmelstjerna FC Mutke MA Bauer M Herzig CX et al . 3D GRASE pulsed arterial spin labeling at multiple inflow times in patients with long arterial transit times: comparison with dynamic susceptibility-weighted contrast-enhanced MRI at 3 tesla . *J Cereb Blood Flow Metab* 2015 ; 35: 392 – 401 . doi: 10.1038/jcbfm.2014.200 25407272 PMC4348376

[25] Sugimori H Fujima N Suzuki Y Hamaguchi H Sakata M Kudo K . Evaluation of cerebral blood flow using multi-phase pseudo continuous arterial spin labeling at 3-tesla . *Magn Reson Imaging* 2015 ; 33: 1338 – 44 : S0730-725X(15)00192-7 . doi: 10.1016/j.mri.2015.07.016 26260545

[26] Johnston ME Lu K Maldjian JA Jung Y . Multi-TI arterial spin labeling MRI with variable TR and bolus duration for cerebral blood flow and arterial transit time mapping . *IEEE Trans Med Imaging* 2015 ; 34: 1392 – 1402 . doi: 10.1109/TMI.2015.2395257 25616010

[27] Yun TJ Sohn CH Yoo RE Kang KM Choi SH Kim JH et al . Transit time corrected arterial spin labeling technique aids to overcome delayed transit time effect . *Neuroradiology* 2018 ; 60: 255 – 65 . doi: 10.1007/s00234-017-1969-x 29288284

[28] Kim HG Lee JH Choi JW Han M Gho SM Moon Y . Multidelay arterial spin-labeling MRI in neonates and infants: cerebral perfusion changes during brain maturation . *AJNR Am J Neuroradiol* 2018 ; 39: 1912 – 18 . doi: 10.3174/ajnr.A5774 30213808 PMC7410720

[29] Zaharchuk G . Arterial transit awesomeness . *Radiology* 2020 ; 297: 661 – 62 . doi: 10.1148/radiol.2020203838 33052076 PMC7706871

[30] Jaganmohan D Pan S Kesavadas C Thomas B . A pictorial review of brain arterial spin labelling artefacts and their potential remedies in clinical studies . *Neuroradiol J* 2021 ; 34: 154 – 68 . doi: 10.1177/1971400920977031 33283653 PMC8165894

[31] Deibler AR Pollock JM Kraft RA Tan H Burdette JH Maldjian JA . Arterial spin-labeling in routine clinical practice, part 1: technique and artifacts . *AJNR Am J Neuroradiol* 2008 ; 29: 1228 – 34 . doi: 10.3174/ajnr.A1030 18372417 PMC4686140

[32] . Amukotuwa SA Yu C Zaharchuk G. 3D Pseudocontinuous arterial spin labeling in routine clinical practice: A review of clinically significant artifacts . J Magn Reson Imaging 2016 ; 43( 1 ): 11 - 27 . 25857715 10.1002/jmri.24873

[33] Bladt P van Osch MJP Clement P Achten E Sijbers J den Dekker AJ . Supporting measurements or more averages? how to quantify cerebral blood flow most reliably in 5 minutes by arterial spin labeling . *Magn Reson Med* 2020 ; 84: 2523 – 36 . doi: 10.1002/mrm.28314 32424947 PMC7402018

[34] Maier O Spann SM Pinter D Gattringer T Hinteregger N Thallinger GG et al . Non-linear fitting with joint spatial regularization in arterial spin labeling . *Medical Image Analysis* 2021 ; 71: 102067 . doi: 10.1016/j.media.2021.102067 33930830

[35] Madai VI Martin SZ von Samson-Himmelstjerna FC Herzig CX Mutke MA Wood CN et al . Correction for susceptibility distortions increases the performance of arterial spin labeling in patients with cerebrovascular disease . *J Neuroimaging* 2016 ; 26: 436 – 44 . doi: 10.1111/jon.12331 26902457

[36] Petr J Schramm G Hofheinz F Langner J van den Hoff J . Modeling magnetization transfer effects of Q2TIPS bolus saturation in multi-TI pulsed arterial spin labeling . *Magn Reson Med* 2014 ; 72: 1007 – 14 . doi: 10.1002/mrm.25011 24194169

[37] Zhang LX Woods JG Okell TW Chappell MA . Examination of optimized protocols for pcasl: sensitivity to macrovascular contamination, flow dispersion, and prolonged arterial transit time . *Magn Reson Med* 2021 ; 86: 2208 – 19 . doi: 10.1002/mrm.28839 34009682 PMC8581991

[38] Kramme J Gregori J Diehl V Madai VI von Samson-Himmelstjerna FC Lentschig M et al . Improving perfusion quantification in arterial spin labeling for delayed arrival times by using optimized acquisition schemes . *Z Med Phys* 2015 ; 25: 221 – 29 : S0939-3889(14)00094-4 . doi: 10.1016/j.zemedi.2014.07.003 25125192

[39] Paschoal AM Leoni RF Foerster BU Dos Santos AC Pontes-Neto OM Paiva FF . Contrast optimization in arterial spin labeling with multiple post-labeling delays for cerebrovascular assessment . *MAGMA* 2021 ; 34: 119 – 31 . doi: 10.1007/s10334-020-00883-z 32885356

[40] Woods JG Chappell MA Okell TW . Designing and comparing optimized pseudo-continuous arterial spin labeling protocols for measurement of cerebral blood flow . *Neuroimage* 2020 ; 223: : S1053-8119(20)30732-1 . doi: 10.1016/j.neuroimage.2020.117246 PMC7762814 32853814

[41] Woods JG Chappell MA Okell TW . A general framework for optimizing arterial spin labeling MRI experiments . *Magn Reson Med* 2019 ; 81: 2474 – 88 . doi: 10.1002/mrm.27580 30588656 PMC6492260

[42] Zaharchuk G . Arterial spin label imaging of acute ischemic stroke and transient ischemic attack . *Neuroimaging Clin N Am* 2011 ; 21: 285 – 301 . doi: 10.1016/j.nic.2011.01.003 21640300 PMC3109302

[43] Guo L Zhang Q Ding L Liu K Ding K Jiang C et al . Pseudo-continuous arterial spin labeling quantifies cerebral blood flow in patients with acute ischemic stroke and chronic lacunar stroke . *Clin Neurol Neurosurg* 2014 ; 125: 229 – 36 : S0303-8467(14)00323-0 . doi: 10.1016/j.clineuro.2014.08.017 25203634

[44] Chen J Zhao B Bu C Xie G . Relationship between the hemodynamic changes on multi-td pulsed arterial spin labeling images and the degrees of cerebral artery stenosis . *Magn Reson Imaging* 2014 ; 32: 1277 – 83 : S0730-725X(14)00248-3 . doi: 10.1016/j.mri.2014.08.017 25171819

[45] Amemiya S Watanabe Y Takei N Ueyama T Miyawaki S Koizumi S et al . Arterial transit time-based multidelay combination strategy improves arterial spin labeling cerebral blood flow measurement accuracy in severe steno-occlusive diseases . *J Magn Reson Imaging* 2022 ; 55: 178 – 87 . doi: 10.1002/jmri.27823 34263988

[46] Wolf ME Layer V Gregori J Griebe M Szabo K Gass A et al . Assessment of perfusion deficits in ischemic stroke using 3D-GRASE arterial spin labeling magnetic resonance imaging with multiple inflow times . *J Neuroimaging* 2014 ; 24: 453 – 59 . doi: 10.1111/jon.12064 25340181

[47] Lou X Yu S Scalzo F Starkman S Ali LK Kim D et al . Multi-delay ASL can identify leptomeningeal collateral perfusion in endovascular therapy of ischemic stroke . *Oncotarget* 2017 ; 8: 2437 – 43 . doi: 10.18632/oncotarget.13898 27974692 PMC5356813

[48] Wang DJJ Alger JR Qiao JX Gunther M Pope WB Saver JL et al . Multi-delay multi-parametric arterial spin-labeled perfusion MRI in acute ischemic stroke - comparison with dynamic susceptibility contrast enhanced perfusion imaging . *Neuroimage Clin* 2013 ; 3: 1 – 7 . doi: 10.1016/j.nicl.2013.06.017 24159561 PMC3791289

[49] Xu X Tan Z Fan M Ma M Fang W Liang J et al . Comparative study of multi-delay pseudo-continuous arterial spin labeling perfusion MRI and CT perfusion in ischemic stroke disease . *Front Neuroinform* 2021 ; 15: 719719 . doi: 10.3389/fninf.2021.719719 34456703 PMC8386683

[50] Amemiya S Takao H Watanabe Y Takei N Ueyama T Kato S et al . Reliability and sensitivity to longitudinal CBF changes in steno-occlusive diseases: ASL versus ^123^ I-imp-SPECT . *J Magn Reson Imaging* 2021 . doi: 10.1002/jmri.27996 34780101

[51] Hendrikse J Petersen ET Golay X . Vascular disorders: insights from arterial spin labeling . *Neuroimaging Clin N Am* 2012 ; 22: 259 – 69 . doi: 10.1016/j.nic.2012.02.003 22548931

[52] Kang JH Yun TJ Yoo RE Yoon BW Lee AL Kang KM et al . Bright sinus appearance on arterial spin labeling MR imaging aids to identify cerebral venous thrombosis . *Medicine (Baltimore*) 2017; 96(41): e8244. 10.1097/MD.0000000000008244 29019892 PMC5662315

[53] Furuya S Kawabori M Fujima N Tokairin K Goto S Iwasaki M et al . Serial arterial spin labeling may be useful in assessing the therapeutic course of cerebral venous thrombosis: case reports . *Neurol Med Chir (Tokyo*) 2017; 57: 557–61. 10.2176/nmc.cr.2017-0033 28835576 PMC5638783

[54] Kronenburg A Bulder MMM Bokkers RPH Hartkamp NS Hendrikse J Vonken E-J et al . Cerebrovascular reactivity measured with ASL perfusion MRI, ivy sign, and regional tissue vascularization in moyamoya . *World Neurosurg* 2019 ; 125: e639 – 50 : S1878-8750(19)30240-2 . doi: 10.1016/j.wneu.2019.01.140 30716498

[55] Zaharchuk G Do HM Marks MP Rosenberg J Moseley ME Steinberg GK . Arterial spin-labeling MRI can identify the presence and intensity of collateral perfusion in patients with moyamoya disease . *Stroke* 2011 ; 42: 2485 – 91 . doi: 10.1161/STROKEAHA.111.616466 21799169 PMC3164217

[56] Ha JY Choi YH Lee S Cho YJ Cheon JE Kim IO et al . Arterial spin labeling MRI for quantitative assessment of cerebral perfusion before and after cerebral revascularization in children with moyamoya disease . *Korean J Radiol* 2019 ; 985 – 96 . 31132824 10.3348/kjr.2018.0651 PMC6536794

[57] Ni WW Christen T Rosenberg J Zun Z Moseley ME Zaharchuk G . Imaging of cerebrovascular reserve and oxygenation in moyamoya disease . *J Cereb Blood Flow Metab* 2017 ; 1213 – 22 . doi: 10.1177/0271678X16651088 PMC5453445 27207169

[58] Federau C Christensen S Zun Z Park SW Ni W Moseley M et al . Cerebral blood flow, transit time, and apparent diffusion coefficient in moyamoya disease before and after acetazolamide . *Neuroradiology* 2017 ; 5 – 12 . 27913820 10.1007/s00234-016-1766-y PMC8006793

[59] Wang R Yu S Alger JR Zuo Z Chen J Wang R et al . Multi-delay arterial spin labeling perfusion MRI in moyamoya disease--comparison with CT perfusion imaging . *Eur Radiol* 2014 ; 24: 1135 – 44 . doi: 10.1007/s00330-014-3098-9 24557051 PMC4143230

[60] Qiu D Straka M Zun Z Bammer R Moseley ME Zaharchuk G . CBF measurements using multidelay pseudocontinuous and velocity-selective arterial spin labeling in patients with long arterial transit delays: comparison with xenon CT CBF . *J Magn Reson Imaging* 2012 ; 110 – 19 . 10.1002/jmri.23613 PMC3368036 22359345

[61] Fan AP Guo J Khalighi MM Gulaka PK Shen B Park JH et al . Long-delay arterial spin labeling provides more accurate cerebral blood flow measurements in moyamoya patients: A simultaneous positron emission tomography/MRI study . *Stroke* 2017 ; 48: 2441 – 49 . doi: 10.1161/STROKEAHA.117.017773 28765286 PMC8006795

[62] Fan AP Khalighi MM Guo J Ishii Y Rosenberg J Wardak M et al . Identifying hypoperfusion in moyamoya disease with arterial spin labeling and an [ ^15^ o]-water positron emission tomography/magnetic resonance imaging normative database . *Stroke* 2019 ; 50: 373 – 80 . doi: 10.1161/STROKEAHA.118.023426 30636572 PMC7161423

[63] Zhao MY Fan AP Chen DY-T Sokolska MJ Guo J Ishii Y et al . Cerebrovascular reactivity measurements using simultaneous ^15^o-water PET and ASL MRI: impacts of arterial transit time, labeling efficiency, and hematocrit . *Neuroimage* 2021 ; 233: S1053-8119(21)00232-9 . doi: 10.1016/j.neuroimage.2021.117955 PMC8272558 33716155

[64] Bulder MMM Bokkers RPH Hendrikse J Kappelle LJ Braun KPJ Klijn CJM . Arterial spin labeling perfusion MRI in children and young adults with previous ischemic stroke and unilateral intracranial arteriopathy . *Cerebrovasc Dis* 2014 ; 37: 14 – 21 . doi: 10.1159/000355889 24355934

[65] Juttukonda MR . Donahue MJ1,2,3,4, davis LT1, gindville MC5, lee CA5, patel NJ5, kassim AA6, pruthi S1, hendrikse J7, jordan LC2,5. preliminary evidence for cerebral capillary shunting in adults with sickle cell anemia . *J Cereb Blood Flow Metab* 2019 ; 39: 1099 – 1110 . 29260615 10.1177/0271678X17746808 PMC6547194

[66] Juttukonda MR Jordan LC Gindville MC Davis LT Watchmaker JM Pruthi S et al . Cerebral hemodynamics and pseudo-continuous arterial spin labeling considerations in adults with sickle cell anemia . *NMR Biomed* 2017 ; 30( 2 . doi: 10.1002/nbm.3681 PMC5351809 28052565

[67] Shen Y Zhao B Yan L Jann K Wang G Wang J et al . Cerebral hemodynamic and white matter changes of type 2 diabetes revealed by multi-TI arterial spin labeling and double inversion recovery sequence . *Front Neurol* 2017 ; 8: 717 . doi: 10.3389/fneur.2017.00717 29312135 PMC5743674

[68] Jia J Xie J Li H Wei H Li X Hu J et al . Cerebral blood flow abnormalities in neuropsychiatric systemic lupus erythematosus . *Lupus* 2019 ; 28: 1128 – 33 . doi: 10.1177/0961203319861677 31315530

[69] Zhuo Z Su L Duan Y Huang J Qiu X Haller S et al . Different patterns of cerebral perfusion in SLE patients with and without neuropsychiatric manifestations . *Hum Brain Mapp* 2020; 41: 755–66. 10.1002/hbm.24837 31650651 PMC7268026

[70] Hoogeveen ES Pelzer N Ghariq E van Osch MJ Dahan A Terwindt GM et al . Cerebrovascular reactivity in retinal vasculopathy with cerebral leukoencephalopathy and systemic manifestations . *J Cereb Blood Flow Metab* 2021 ; 41: 831 – 40 . doi: 10.1177/0271678X20929430 33736510 PMC7983338

[71] Kayfan S Sharifi A Xie S Yin C Pfeifer CM . MRA and ASL perfusion findings in pediatric reversible cerebral vasoconstriction syndrome . *Radiol Case Rep* 2019 ; 14: 832 – 36 . doi: 10.1016/j.radcr.2019.04.010 31061686 PMC6487463

[72] Wakisaka K Morioka T Shimogawa T Murao K Kanazawa Y Hagiwara N et al . Epileptic ictal hyperperfusion on arterial spin labeling perfusion and diffusion-weighted magnetic resonance images in posterior reversible encephalopathy syndrome . *J Stroke Cerebrovasc Dis* 2016 ; 25: 228 – 37 : S1052-3057(15)00509-1 . doi: 10.1016/j.jstrokecerebrovasdis.2015.09.023 26515648

[73] Huang BY Castillo M . Hypoxic-ischemic brain injury: imaging findings from birth to adulthood . *Radiographics* 2008 ; 28: 417 – 39 . doi: 10.1148/rg.282075066 18349449

[74] Mangla R Kolar B Almast J Ekholm SE . Border zone infarcts: pathophysiologic and imaging characteristics . *Radiographics* 2011 ; 31: 1201 – 14 . doi: 10.1148/rg.315105014 21918038

[75] Zaharchuk G Bammer R Straka M Shankaranarayan A Alsop DC Fischbein NJ et al . Arterial spin-label imaging in patients with normal bolus perfusion-weighted MR imaging findings: pilot identification of the borderzone sign . *Radiology* 2009 ; 252: 797 – 807 . doi: 10.1148/radiol.2523082018 19703858 PMC6939961

[76] Iordanova B1, li L2, clark RSB3,4, manole MD3. alterations in cerebral blood flow after resuscitation from cardiac arrest . *Front Pediatr* 2017 ; 5: 174 . doi: 10.3389/fped.2017.00174 28861407 PMC5561008

[77] Li N Wingfield MA Nickerson JP Pettersson DR Pollock JM . Anoxic brain injury detection with the normalized diffusion to ASL perfusion ratio: implications for blood-brain barrier injury and permeability . *AJNR Am J Neuroradiol* 2020 ; 41: 598 – 606 . doi: 10.3174/ajnr.A6461 32165356 PMC7144662

[78] Wong AM-C Yeh C-H Liu H-L Wu T-W Lin K-L Wang H-S et al . Arterial spin-labeling perfusion imaging of children with subdural hemorrhage: perfusion abnormalities in abusive head trauma . *J Neuroradiol* 2017 ; 44: 281 – 87 : S0150-9861(16)30135-3 . doi: 10.1016/j.neurad.2017.02.003 28341000

[79] Prosser DD Grigsby T Pollock JM . Unilateral anoxic brain injury secondary to strangulation identified on conventional and arterial spin-labeled perfusion imaging . *Radiol Case Rep* 2018 ; 13: 563 – 67 . doi: 10.1016/j.radcr.2018.02.004 29988732 PMC6030549

[80] Tortora D Severino M Rossi A . Arterial spin labeling perfusion in neonates . *Semin Fetal Neonatal Med* 2020 ; 25: 101130 : S1744-165X(20)30055-X . doi: 10.1016/j.siny.2020.101130 32591228

[81] Kim HG Choi JW Lee JH Jung DE Gho SM . Association of cerebral blood flow and brain tissue relaxation time with neurodevelopmental outcomes of preterm neonates: multidelay arterial spin labeling and synthetic MRI study . *Invest Radiol* 2022 ; 57: 254 – 62 . doi: 10.1097/RLI.0000000000000833 34743135

[82] Bouyssi-Kobar M Murnick J Brossard-Racine M Chang T Mahdi E Jacobs M et al . Altered cerebral perfusion in infants born preterm compared with infants born full term . *J Pediatr* 2018 ; 193: 54 - 61 . S0022-3476(17)31338-0 . doi: 10.1016/j.jpeds.2017.09.083 29212618 PMC5794508

[83] Miranda MJ Olofsson K Sidaros K . Noninvasive measurements of regional cerebral perfusion in preterm and term neonates by magnetic resonance arterial spin labeling . *Pediatr Res* 2006 ; 60: 359 – 63 . doi: 10.1203/01.pdr.0000232785.00965.b3 16857776

[84] Watson CG Dehaes M Gagoski BA Grant PE Rivkin MJ . Arterial spin labeling perfusion magnetic resonance imaging performed in acute perinatal stroke reveals hyperperfusion associated with ischemic injury . *Stroke* 2016 ; 47: 1514 – 19 . doi: 10.1161/STROKEAHA.115.011936 27143277

[85] Benninger KL Peng J Ho ML Newton J Wang DJJ Hu HH et al . Cerebral perfusion and neurological examination characterise neonatal opioid withdrawal syndrome: a prospective cohort study . *Arch Dis Child Fetal Neonatal Ed* 2021 : fetalneonatal-2021-322192 . doi: 10.1136/archdischild-2021-322192 34725106

[86] Scheffer IE Berkovic S Capovilla G Connolly MB French J Guilhoto L et al . ILAE classification of the epilepsies: position paper of the ILAE commission for classification and terminology . *Epilepsia* 2017 ; 58: 512 – 21 . doi: 10.1111/epi.13709 28276062 PMC5386840

[87] Fisher RS Cross JH French JA Higurashi N Hirsch E Jansen FE et al . Operational classification of seizure types by the international league against epilepsy: position paper of the ILAE commission for classification and terminology . *Epilepsia* 2017 ; 58: 522 – 30 . doi: 10.1111/epi.13670 28276060

[88] Pressler RM Cilio MR Mizrahi EM Moshé SL Nunes ML Plouin P et al . ( n.d .). The ILAE classification of seizures and the epilepsies: modification for seizures in the neonate . *Position Paper by the ILAE Task Force on Neonatal Seizures Epilepsia* ; 62: 615 – 28 . doi: 10.1111/epi.16815 33522601

[89] Blauwblomme T Boddaert N Chémaly N Chiron C Pages M Varlet P et al . Arterial spin labeling MRI: a step forward in non-invasive delineation of focal cortical dysplasia in children . *Epilepsy Res* 2014 ; 108: 1932 – 39 : S0920-1211(14)00272-1 . doi: 10.1016/j.eplepsyres.2014.09.029 25454505

[90] Zeng JY Hu XQ Xu JF Zhu WJ Wu HY Dong FJ . Diagnostic accuracy of arterial spin-labeling MR imaging in detecting the epileptogenic zone: systematic review and meta-analysis . *AJNR Am J Neuroradiol* 2021 ; 42: 1052 – 60 . doi: 10.3174/ajnr.A7061 33766822 PMC8191675

[91] Nagesh C1, kumar S1, menon R2, thomas B1, radhakrishnan A2, kesavadas C1. the imaging of localization related symptomatic epilepsies: the value of arterial spin labelling based magnetic resonance perfusion . *Korean J Radiol* 2018 ; 965 – 77 . doi: 10.3348/kjr.2018.19.5.965 30174487 PMC6082755

[92] Oner AY Eryurt B Ucar M Capraz I Kurt G Bilir E et al . PASL versus DSC perfusion MRI in lateralizing temporal lobe epilepsy . *Acta Radiol* 2015 ; 56: 477 – 81 . doi: 10.1177/0284185114531128 24782571

[93] Pizzini FB Farace P Manganotti P Zoccatelli G Bongiovanni LG Golay X et al . Cerebral perfusion alterations in epileptic patients during peri-ictal and post-ictal phase: PASL vs DSC-MRI . *Magn Reson Imaging* 2013 ; 31: 1001 – 5 : S0730-725X(13)00109-4 . doi: 10.1016/j.mri.2013.03.023 23623332

[94] Takane Y Shibata K Nishimura Y Sakura H . Crossed cerebellar and contralateral thalamic hyperperfusion in epilepsy . *Intern Med* 2017 ; 56: 1121 – 22 . doi: 10.2169/internalmedicine.56.7632 28458326 PMC5478581

[95] Chen G Lei D Ren J Zuo P Suo X Wang DJJ et al . Patterns of postictal cerebral perfusion in idiopathic generalized epilepsy: a multi-delay multi-parametric arterial spin labelling perfusion MRI study . *Sci Rep* 2016 ; 6( 1 . doi: 10.1038/srep28867 PMC4931466 27374369

[96] Kim TJ Choi JW Han M Kim BG Park SA Huh K et al . Usefulness of arterial spin labeling perfusion as an initial evaluation of status epilepticus . *Sci Rep* 2021 ; 11( 1 ): 24218 . doi: 10.1038/s41598-021-03698-7 34930959 PMC8688435

[97] Cadiot D Longuet R Bruneau B Treguier C Carsin-Vu A Corouge I et al . Magnetic resonance imaging in children presenting migraine with aura: association of hypoperfusion detected by arterial spin labelling and vasospasm on MR angiography findings . *Cephalalgia* 2018 ; 38: 949 – 58 . doi: 10.1177/0333102417723570 28738690

[98] Cobb-Pitstick KM Munjal N Safier R Cummings DD Zuccoli G . Time course of cerebral perfusion changes in children with migraine with aura mimicking stroke . *AJNR Am J Neuroradiol* 2018 ; 39: 1751 – 55 . doi: 10.3174/ajnr.A5693 29903927 PMC7655279

[99] Wolf ME Okazaki S Eisele P Rossmanith C Gregori J Griebe M et al . Arterial spin labeling cerebral perfusion magnetic resonance imaging in migraine aura: an observational study . *J Stroke Cerebrovasc Dis* 2018 ; 27: 1262 – 66 : S1052-3057(17)30664-X . doi: 10.1016/j.jstrokecerebrovasdis.2017.12.002 29331612

[100] Kato Y Araki N Matsuda H Ito Y Suzuki C . Arterial spin-labeled MRI study of migraine attacks treated with rizatriptan . *J Headache Pain* 2010 ; 11: 255 – 58 . doi: 10.1007/s10194-010-0215-2 20411294 PMC3451919

[101] Andre JB . Arterial spin labeling magnetic resonance perfusion for traumatic brain injury: technical challenges and potentials . *Top Magn Reson Imaging* 2015 ; 24: 275 – 87 . doi: 10.1097/RMR.0000000000000065 26502309

[102] Kikuchi K Hiwatashi A Togao O Yamashita K Yoshimoto K Mizoguchi M et al . Correlation between arterial spin-labeling perfusion and histopathological vascular density of pediatric intracranial tumors . *J Neurooncol* 2017 ; 135: 561 – 69 . doi: 10.1007/s11060-017-2604-8 28856499

[103] Delgado AF De Luca F Hanagandi P van Westen D Delgado AF . Arterial spin-labeling in children with brain tumor: A meta-analysis . *AJNR Am J Neuroradiol* 2018 ; 39: 1536 – 42 . doi: 10.3174/ajnr.A5727 30072368 PMC7410530

[104] Vidyasagar R Abernethy L Pizer B Avula S Parkes LM . Quantitative measurement of blood flow in paediatric brain tumours-a comparative study of dynamic susceptibility contrast and multi time-point arterial spin labelled MRI . *Br J Radiol* 2016 ; 89( 1062 ): 20150624 . doi: 10.1259/bjr.20150624 26975495 PMC5258143

[105] Yang S Zhao B Wang G Xiang J Xu S Liu Y et al . Improving the grading accuracy of astrocytic neoplasms noninvasively by combining timing information with cerebral blood flow: A multi-TI arterial spin-labeling MR imaging study . *AJNR Am J Neuroradiol* 2016 ; 37: 2209 – 16 . doi: 10.3174/ajnr.A4907 27561831 PMC7963858

[106] Maral H Ertekin E Tunçyürek Ö Özsunar Y . Effects of susceptibility artifacts on perfusion MRI in patients with primary brain tumor: A comparison of arterial spin-labeling versus DSC . *AJNR Am J Neuroradiol* 2020 ; 41: 255 – 61 . doi: 10.3174/ajnr.A6384 31974077 PMC7015218

[107] Noguchi T Yakushiji Y Nishihara M Togao O Yamashita K Kikuchi K et al . Arterial spin-labeling in central nervous system infection . *Magn Reson Med Sci* 2016 ; 15: 386 – 94 . doi: 10.2463/mrms.mp.2015-0140 27001393 PMC5608113

[108] Kumar S Gutch M . Advanced magnetic resonance imaging techniques in tuberculous meningitis . *Adv Biomed Res* 2020 ; 9: 20 . doi: 10.4103/abr.abr_222_19 32695730 PMC7365389

[109] Zhang X . Guo X1, zhang N1, cai H2, sun J1, wang Q1, qi Y3, zhang L3, yang L3, shi FD3, yu C1. cerebral blood flow changes in multiple sclerosis and neuromyelitis optica and their correlations with clinical disability . *Front Neurol* 2018 . doi: 10.3389/fneur.2018.00305 PMC5946009 29780351

[110] Ferreira CR Rahman S Keller M Zschocke J Group IA . An international classification of inherited metabolic disorders (ICIMD) . *J Inherit Metab Dis* 2021 ; 44: 164 – 77 . doi: 10.1002/jimd.12348 33340416 PMC9021760

[111] Li X Wang Y Wang Z Lu J Xu Y Ye J et al . Comparison of magnetic resonance spectroscopy (MRS) with arterial spin labeling (ASL) in the differentiation between mitochondrial encephalomyopathy, lactic acidosis, plus stroke-like episodes (melas) and acute ischemic stroke (ais) . *J Clin Neurosci* 2018; 55: 65–70: S0967-5868(17)31727-7. 10.1016/j.jocn.2018.06.015 29921486

[112] Ikawa M Yoneda M Muramatsu T Matsunaga A Tsujikawa T Yamamoto T et al . Detection of preclinically latent hyperperfusion due to stroke-like episodes by arterial spin-labeling perfusion MRI in MELAS patients . *Mitochondrion* 2013 ; 13: 676 – 80 : S1567-7249(13)00251-1 . doi: 10.1016/j.mito.2013.09.007 24095972

[113] Whitehead MT Lee B Gropman A . Lesional perfusion abnormalities in leigh disease demonstrated by arterial spin labeling correlate with disease activity . *Pediatr Radiol* 2016 ; 46: 1309 – 16 . doi: 10.1007/s00247-016-3616-9 27043731

[114] Phyu P Merwick A Davagnanam I Bolsover F Jichi F Wheeler-Kingshott C et al . Increased resting cerebral blood flow in adult fabry disease: MRI arterial spin labeling study . *Neurology* 2018 ; 90: e1379 – 85 . doi: 10.1212/WNL.0000000000005330 29661900 PMC5902785

[115] Sekar S Vinayagamani S Thomas B Kesavadas C . Arterial spin labeling hyperperfusion in seizures associated with non-ketotic hyperglycaemia: is it merely a post-ictal phenomenon? *Neurol Sci* 2021 ; 42: 739 – 44 . doi: 10.1007/s10072-020-04815-6 33047197

